# Low-level blast exposure induces chronic vascular remodeling, perivascular astrocytic degeneration and vascular-associated neuroinflammation

**DOI:** 10.1186/s40478-021-01269-5

**Published:** 2021-10-15

**Authors:** Miguel A. Gama Sosa, Rita De Gasperi, Dylan Pryor, Georgina S. Perez Garcia, Gissel M. Perez, Rania Abutarboush, Usmah Kawoos, Seth Hogg, Benjamin Ache, William G. Janssen, Allison Sowa, Timothy Tetreault, David G. Cook, Susan J. Tappan, Sam Gandy, Patrick R. Hof, Stephen T. Ahlers, Gregory A. Elder

**Affiliations:** 1 General Medical Research Service, James J. Peters Department of Veterans Affairs Medical Center, 130 West Kingsbridge Road, Bronx, NY 10468 USA; 2 Department of Psychiatry, Icahn School of Medicine at Mount Sinai, One Gustave Levy Place, New York, NY 10029 USA; 3 Friedman Brain Institute, Icahn School of Medicine at Mount Sinai, New York, NY 10029 USA; 4 Research and Development Service, James J. Peters Department of Veterans Affairs Medical Center, 130 West Kingsbridge Road, Bronx, NY 10468 USA; 5 Department of Neurology, Icahn School of Medicine at Mount Sinai, One Gustave Levy Place, New York, NY 10029 USA; 6 Nash Family Department of Neuroscience, Icahn School of Medicine at Mount Sinai, New York, NY 10029 USA; 7 Department of Neurotrauma, Operational and Undersea Medicine Directorate, Naval Medical Research Center, 503 Robert Grant Avenue, Silver Spring, MD 20910 USA; 8 The Henry M. Jackson Foundation for the Advancement of Military Medicine Inc, Bethesda, MD USA; 9 Micro Photonics, Inc, 1550 Pond Road, Suite 110, Allentown, PA 18104 USA; 10 MBF Bioscience LLC, 185 Allen Brook Lane, Williston, VT 05495 USA; 11 Geriatric Research Education and Clinical Center, VA Puget Sound Health Care System, 1660 S Columbian Way, Seattle, WA 98108 USA; 12 Department of Medicine, University of Washington, 1959 NE Pacific St, Seattle, WA 98195 USA; 13 Mount Sinai Alzheimer’s Disease Research Center and the Ronald M. Loeb Center for Alzheimer’s Disease, Icahn School of Medicine at Mount Sinai, New York, NY 10029 USA; 14 NFL Neurological Care Center, Icahn School of Medicine at Mount Sinai, New York, NY 10029 USA; 15 Department of Geriatrics and Palliative Care, Icahn School of Medicine at Mount Sinai, New York, NY 10029 USA; 16 Neurology Service, James J. Peters Department of Veterans Affairs Medical Center, 130 West Kingsbridge Road, Bronx, NY 10468 USA

**Keywords:** Animal model, Blast, Brain, Chronic, Vascular, Neurovascular unit, Rat, Vascular pathology, Astrocyte, Tight junctions

## Abstract

Cerebral vascular injury as a consequence of blast-induced traumatic brain injury is primarily the result of blast wave-induced mechanical disruptions within the neurovascular unit. In rodent models of blast-induced traumatic brain injury, chronic vascular degenerative processes are associated with the development of an age-dependent post-traumatic stress disorder-like phenotype. To investigate the evolution of blast-induced chronic vascular degenerative changes, Long-Evans rats were blast-exposed (3 × 74.5 kPa) and their brains analyzed at different times post-exposure by X-ray microcomputed tomography, immunohistochemistry and electron microscopy. On microcomputed tomography scans, regional cerebral vascular attenuation or occlusion was observed as early as 48 h post-blast, and cerebral vascular disorganization was visible at 6 weeks and more accentuated at 13 months post-blast. Progression of the late-onset pathology was characterized by detachment of the endothelial and smooth muscle cellular elements from the neuropil due to degeneration and loss of arteriolar perivascular astrocytes. Development of this pathology was associated with vascular remodeling and neuroinflammation as increased levels of matrix metalloproteinases (MMP-2 and MMP-9), collagen type IV loss, and microglial activation were observed in the affected vasculature. Blast-induced chronic alterations within the neurovascular unit should affect cerebral blood circulation, glymphatic flow and intramural periarterial drainage, all of which may contribute to development of the blast-induced behavioral phenotype. Our results also identify astrocytic degeneration as a potential target for the development of therapies to treat blast-induced brain injury.

## Introduction

Traumatic brain injury (TBI) has been linked to mental health disorders and is considered a risk factor for later development of neurodegenerative disorders [31]. Many veterans from the recent conflicts in Iraq and Afghanistan suffer from chronic neurobehavioral syndromes that include post-traumatic stress disorder (PTSD) [31]. Indeed, a striking feature in veterans from the most recent war theaters has been the overlap between a history of blast-related mild TBI (mTBI) and PTSD [17, 31, 115, 134, 140, 141]. While these symptoms may improve, they frequently persist and may worsen with declines driven mainly by worsening PTSD and depression [83]. Although the mechanisms underlying how blast affects human neurobiology are incompletely understood, much evidence suggests that the cerebrovasculature may be particularly vulnerable to blast [112, 131, 133].

Research in rat models of repetitive low level blast exposure has shown that animals exposed to repetitive low-level blast exhibit chronic cognitive impairment and PTSD-related traits that develop over time [30, 33, 100, 101, 123]. These traits include anxiety, enhanced acoustic startle, altered fear learning and impaired cognition in tests such as novel object recognition. Blast-exposed rats thus model many of the features found in human PTSD. A single predator scent challenge delivered 8 months after the last blast exposure induces additional anxiety-related changes that are still present 45 days later [101] suggesting that besides inducing PTSD-related traits blast exposure sensitizes the brain to react abnormally to subsequent psychological stressors.

The evolution of the resulting blast-induced cognitive and behavioral phenotypes in rats seems to overlap the development of cerebral vascular degenerative processes. [38–40]. Cerebral blood circulation is essential for normal brain metabolic functions, as it provides oxygen, glucose and other essential metabolites as well as removes metabolic wastes [125]. Increased synaptic activity increases blood flow in brain regions where higher oxygen concentrations are required via neurovascular coupling. The neurovascular unit is composed of endothelial cells, associated blood–brain barrier tight junctions, a basal lamina covered with mural cells (pericytes and smooth muscle), neural cells (astrocytes and neurons) and an extracellular matrix (ECM). The neurovascular unit also encompasses the glymphatic and intramural periarterial drainage systems. The glymphatic CSF waste clearance system utilizes perivascular channels, formed by astroglial cells, to promote elimination of soluble proteins and metabolites from the CNS [14, 61, 91]. In this sytem, CSF enters interstitial spaces after aquaporin 4 (AQP4)-dependent transport through the astroglial cytoplasm, drains into perivenous routes in a direction parallel to the blood flow, and then enters the subarachnoid CSF or bloodstream across cerebral vessels [142]. The intramural periarterial drainage system (IPAD) is a major pathway by which solutes drain from the brain to cervical lymph nodes along the walls of cerebral arteries in a direction counter to the blood flow [1, 56, 136]. In the IPAD system, parenchymal interstitial fluid (ISF) enters the basal membrane of capillaries and is progressively moved onto the basal membrane of the tunica media of intracerebral arterioles and arteries, and onto the walls of leptomeningeal and internal carotid arteries[5, 19, 142].

Direct consequences of blast exposure include acute damage and chronic structural degeneration of the cerebral vasculature. Early blast-induced vascular injury includes apoptosis of vascular structural elements, capillary strictures, vascular occlusion, blood–brain barrier disruption, vascular rupture, breakdown of the choroid plexus, reduced dilator responses to decreased intravascular pressure, reduced cerebral perfusion, and increased cerebral vascular resistance, [38, 39, 63, 66, 75, 113, 114]. These acute events are followed by the development of a chronic secondary pathology characterized by perivascular astrocytic degeneration, luminal collapse, disruption of neurovascular interactions, ECM remodelling, “double-barreled” vessels, intraluminal astrocytic processes, vascular smooth muscle degeneration, vascular occlusion by CD34^+^ progenitor cells, generalized vascular attenuation, aneurysm formation, vascular fragility and stroke [40]. These combined events can lead to disruptions in cerebral blood circulation that seem to be associated with development of PTSD-related symptoms in blast-exposed individuals [31, 33, 83, 102, 132]. Similarly, CSF and ISF drainage in the brain through the glymphatic and IPAD systems along the external perivascular spaces and laminae may also be affected.

In the present research, we used a combination of X-ray microcomputed tomography (micro-CT) with computerized morphological analyses to determine the evolution of vascular structural alterations in the brain circulation of blast-exposed rats. Micro-CT scans revealed regional cerebral vascular attenuation as early as 48 h post-blast and cerebral vascular attenuation and disorganization at 6 weeks and 13 months post-blast. Immunohistochemical and histological analyses confirmed the presence of previously reported vascular degenerative processes but also revealed at 13 months post-blast the emergence of a vascular pathology characterized by the detachment of the vasculature from the brain parenchyma with affected arterioles at the core of an enlarged paravascular space. This chronic vascular pathology was associated with perivascular-astrocytic degeneration, vascular attenuation, vascular-associated inflammation and vascular remodeling. Our results further document the evolution of blast-induced neurovascular uncoupling due to astrocytic and vascular degenerative processes and provide evidence for a novel pathology that may affect blood circulation and perivascular flow of CSF and ISF through the glymphatic and IPAD systems in the brain.

## Materials and methods

### Animals

Adult male Long-Evans hooded rats (250–350 g, 10 weeks of age, Charles River Laboratories International, Wilmington, MA, USA) were used. All studies involving animals were reviewed and approved by the Institutional Animal Care and Use Committees of the Walter Reed Army Institute of Research (WRAIR)/Naval Medical Research Center and the James J. Peters VA Medical Center. Studies were conducted in compliance with the Public Health Service policy on the humane care and use of laboratory animals, the NIH Guide for the Care and Use of Laboratory Animals, and all applicable Federal regulations governing the protection of animals in research.

### Blast overpressure exposure

The Walter Reed Army Institute of Research (WRAIR) shock tube at the Naval Medical Research Center (NMRC, Silver Springs, MD, USA) was used to expose rats to overpressure injury. This apparatus, which simulates the effects of air blast exposure under experimental conditions, has been used in our prior studies to deliver blast overpressure injury to rats [4, 20–22, 25, 30, 39, 40]. As in previous studies, anesthetized rats were randomly assigned to sham or blast conditions with the head facing the blast exposure without any body shielding, resulting in a full body exposure to the blast wave. The physical characteristics of the blast wave and further details of the blast exposure have been described in detail [4]. Blast-exposed animals received a total of three 74.5-kPa (10.8 psi) exposures, with one exposure administered daily for 3 consecutive days. Control animals were anesthetized and placed in the blast tube but not subjected to a blast exposure. Within 10 days after the last blast exposure, the animals were transferred to the James J. Peters VA Medical Center (Bronx, NY, USA) where all other procedures were performed. Control and experimental cohorts were euthanized at 48 h (n = 6 per group), 6 weeks (n = 6 per group) and 13 months (n = 4 blast-exposed rats and n = 5 control rats) post-blast for observation of acute, subacute and chronic effects, respectively.

### Behavioral testing

Some animals used in this study were previously characterized behaviorally [99]. Data from selected animals were used here. Elevated zero maze (EZM) as well as contextual and cued fear conditioning (FC) tests were performed at 40 and 42 weeks post-exposure, respectively [99].

### X-ray high-resolution micro-CT scanning

Rats were anesthetized with 150 mg/kg ketamine and 30 mg/kg xylazine and transcardially perfused first with 60 ml of 10 µg/ml heparin in phosphate-buffered saline (PBS), pH 7.2, followed by 250 ml of a 30% solution of the Brite Vu Special Projects contrast agent supplemented with its enhancer (Scarlet Imaging, Murray UT, USA) maintained at 65 °C. The perfused animals were chilled by immersion in an ice-water bath for 2 h to gel the intravascular contrast agent. Brains were then dissected, post-fixed overnight in 4% paraformaldehyde in PBS and maintained in sterile PBS at 4 °C. Brains were scanned at a 7.5-µm voxel size using 60 kV, 166 µA X-ray settings and a 0.25-mm aluminum filter with exposure time of 508 ms per frame, with 3 frames averaged at each projection angle with a Bruker SkyScan 1272 micro-CT (Micro Photonics, Allentown, PA, USA). Three-dimensional reconstruction and morphological profiling of the cerebral vasculature was performed with the Bruker’s NRecon software or the Vesselucida 360 software (v2018.1.1, MBF Bioscience LLC, Williston, VT, USA) using data obtained from the micro-CT scans and reconstructing the respective three-dimensional vascular networks. For quantitative analyses, automatic reconstruction of the vasculature with the Vesselucida 360 software was performed using identical settings for all animals. A voxel scooping algorithm was applied with the following settings: trace and seed sensitivity set to 80, medium seed density with refine filter set to 2, maximum gap tolerance. No manual editing was performed. The parameters determined were total vascular length, volume, surface area, length density, the number of isolated vessels, the longest vessel, the number of branching nodes, number of ending vessels and the estimated total tissue volume.

### Histological and immunohistochemical analysis

Coronal sections (50 µm thickness) of the micro-CT scanned brains were prepared with a VT1000S Vibratome (Leica Biosystems, Buffalo Grove, IL, USA). General histology was assessed by hematoxylin–eosin staining of sections. For immunohistochemistry, sections were blocked with 10% normal goat serum in 50 mM Tris HCl, pH 7.6, 0.15 M NaCl, 0.3% Triton-X-100 and incubated overnight with the primary antibodies diluted in blocking solution at room temperature. After washing with PBS (6 times for 10 min each), sections were incubated with the appropriate Alexa (488, 568 and 647)-conjugated secondary antibodies (1:300, ThermoFisher, Waltham, MA, USA) in blocking solution for 2 h. After washing with PBS (6 times for 10 min each), the sections were mounted with Fluorogel mounting medium (Electron Microscopy Sciences, Hatfield, PA, USA). To visualize nuclei, sections were incubated in PBS containing 0.1 µg/ml DAPI (4',6-diamidine-2’-phenylindole dihydrochloride) in the next to last PBS wash. The primary antibodies were a rat monoclonal anti-glial fibrillary acidic protein (GFAP; 1:500, clone 2.2B10, gift of Dr. Virginia Lee, University of Pennsylvania, Philadelphia PA, USA), rabbit anti-GFAP (1:500, G9269, RRID: AB_477035, Sigma-Aldrich, St Louis, MO, USA), mouse anti-α-smooth muscle actin (αSMA, 1:300, A2547, RRID: AB_262054, Sigma-Aldrich), rabbit monoclonal anti-MMP-9 (1:300, 13667S, RRID: AB_2798289, Cell Signaling Technology, Danvers, MA, USA), mouse monoclonal anti-MMP-9 (1:200, NBP2-80855, RRID: AB_2811297, Novus, Littleton, CO, USA) and rabbit anti‐ionized calcium‐binding adaptor molecule 1 (Iba1; 019-19741, RRID: AB_839504, Fujifilm Wako Pure Chemical, Osaka, Japan). Vascular staining was performed with anti-collagen type IV antibodies (rabbit anti-rat collagen type IV, 1:300, ab6586, RRID: AB_305584, Abcam, Cambridge, MA, USA) either in the absence or presence of a pepsin treatment [37, 42]. For the pepsin treatment, sections were incubated with 1 mg/ml pepsin (Agilent Technologies, Santa Clara, CA, USA) in 3% acetic acid for 50 min at 37 °C. Sections were washed once in 0.1 M sodium borate, pH 8.5, washed 5 times with PBS, blocked and stained with antibodies against collagen IV and GFAP in combination with the respective Alexa-conjugated secondary antibodies as described above. For TUNEL staining (terminal deoxynucleotidyltransferase-mediated dUTP nick-end labeling), sections were washed in TBS, permeabilized with 0.1% Triton X-100 in TBS for 1 h and washed extensively with TBS. End labeling of DNA with fluorescein-dUTP was performed using a commercial kit (Roche, Indianapolis, IN, USA). After several washes with PBS, the sections were blocked and stained with antibodies against GFAP and ionized calcium-binding adaptor molecule (Iba1) as described above.

### Electron microscopy

Electron microscopy was performed using protocols optimized to study the ultrastructure of the vasculature. Anesthetized rats were transcardially perfused with ice-cold 2.0% glutaraldehyde, 2% paraformaldenyde, 0.1 M sodium phosphate (pH 7.3). Brains were removed, postfixed in the same fixative as above and stored at 4 °C until ready for processing. Fixed brains were placed on a rat brain slicer matrix, and coronal slices containing the frontal cortex were excised. Sections were washed in 0.1 M sodium cacodylate buffer, pH 7.2 and postfixed with 1% osmium tetroxide in 0.1 M sodium cacodylate buffer, pH 7.2. Sections were washed again in cacodylate buffer, dehydrated through graduated ethanol (70–100%) and propylene oxide series, and resin-infiltrated with Epon (Electron Microscopy Sciences, Hatfield, PA, USA). Material was polymerized in a vacuum oven at 60 °C for 48 h. Semi-thin (1 µm) Toluidine blue-stained sections were used to identify the regions of interest. Ultrathin sections (80 nm) were cut with a diamond knife on a Leica UCT ultramicrotome and mounted on copper grids using a Coat-Quick adhesive pen (Electron Microscopy Sciences). Sections were counterstained with uranyl acetate and lead citrate. Frontal cortical sections were imaged on a Hitachi 7700 electron microscope (Hitachi, LTD., Tokyo, Japan) and photographed with an Advantage CCD camera (Advanced Microscopy Techniques, Danvers, MA, USA). Image brightness and contrast were adjusted using Adobe Photoshop 2021 software (version 22.3.1; Adobe, Inc., San Jose, CA, USA).

### Zymography of vascular metalloproteases

Enriched cerebral vascular fractions were prepared as previously described [40]. Blast-exposed and control rats (6 weeks post-blast) were euthanized by CO_2_ inhalation. Brains were cleaned of meninges and homogenized in cold 18% dextran in PBS (10 ml/g of tissue) using a Potter-Elvejehm homogenizer with a loose-fit Teflon pestle (6–8 strokes at low speed, setting 2 of a Wheaton overhead stirrer, Millville, NJ, USA). Each homogenate was overlayed over an equal volume of Ficoll-Paque PLUS™ (GE Healthcare Life Sciences, Marlborough, MA, USA) and centrifuged for 30 min at 1500×g and 4 °C. Vascular-enriched pellets were resuspended in PBS, washed twice with PBS, and stored at -80 °C [40]. Enriched vascular fractions were lysed in 10 mM NaPO_4_, pH 7.4, 150 mM NaCl, 2 mM EDTA, 1% Triton X-100, 0.5% sodium deoxycholate, and 1% sodium dodecyl sulfate (SDS) supplemented with protease and phosphatase inhibitor cocktails 2 and 3 (Sigma-Aldrich, St Louis, MO, USA). Lysates were centrifuged at 15,000×g for 15 min, and the protein concentration in the supernatants was determined with the BCA reagent (ThermoFisher, Waltham MA, USA). Heat-denatured samples containing 20 µg of protein were electrophoresed through a 10% polyacrylamide gel containing 0.1% gelatin (Novex 10% Zymogram Plus Gelatin Protein Gel, Thermo Fisher Scientific, Waltham, MA). After protein renaturation, incubation, and staining with Brilliant Blue, gelatinase activity was visualized as cleared bands reflective of gelatin hydrolysis.

### Statistical analyses

Statistical differences were evaluated with unpaired *t*-tests using Prism 7.0 software (GraphPad, La Jolla, CA). Statistical significance was set at an α level of 0.05.

## Results

### Cerebral vascular disorganization revealed by micro-CT scanning

**Fig. 1:**
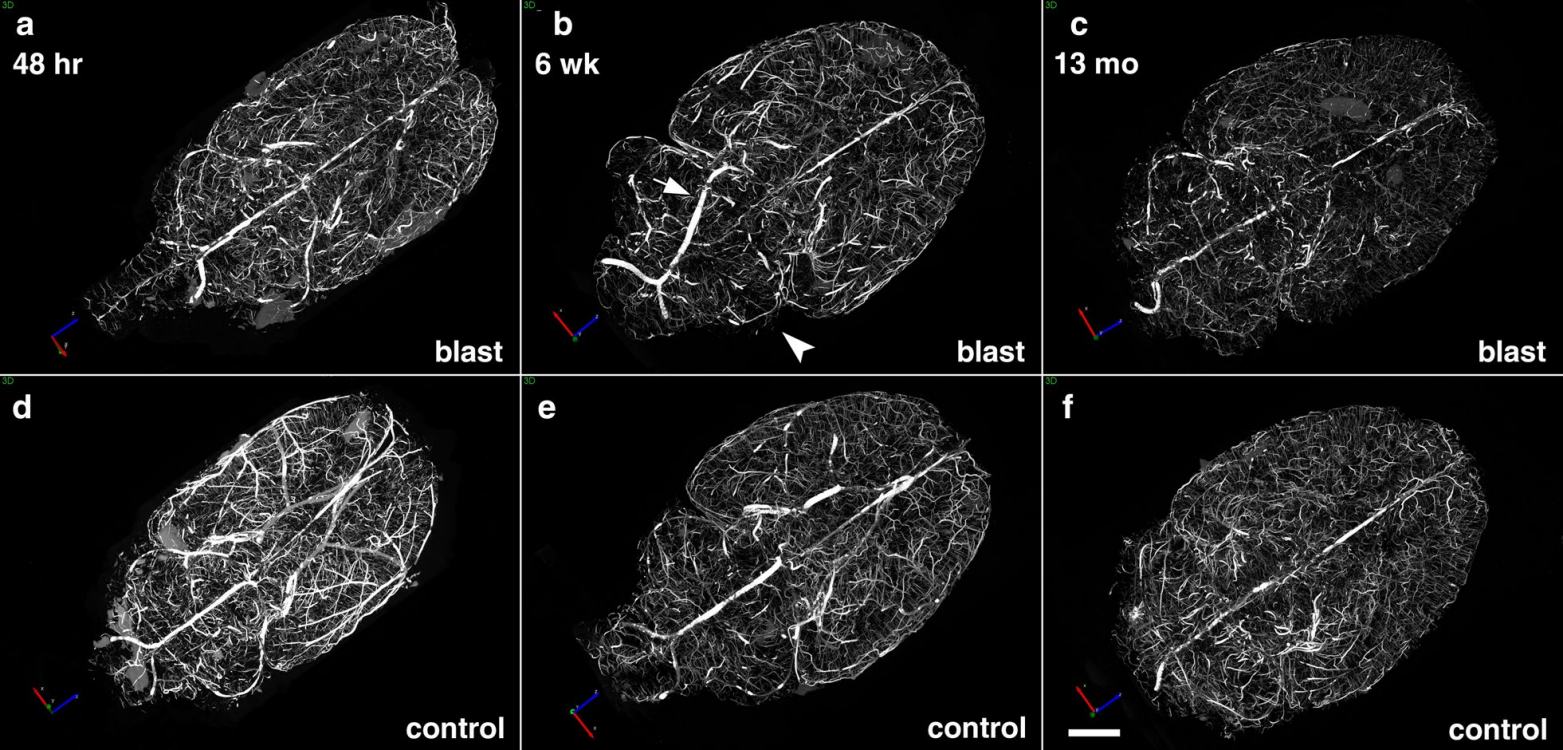
Micro-CT scanning of brains of blast-exposed and control rats. Blast-exposed and control rats were transcardially perfused with the Brite Vu contrast agent at 48 h, 6 weeks and 13 months after blast exposure. Brains were scanned at a resolution of 7.5 µm using 0.3° rotational steps of view around 360° and three-dimensional reconstructions were prepared with Bruker’s NRecon software before visualization with Bruker’s CTVox 3D visualization software. Representative maximum intensity projection (MIP) images show dorsal view of volume-rendered brain vasculature from blast-exposed (**a**–**c**) and control (**d**–**f**) rats. Arrow in **b** shows an apparently displaced basilar artery in this blast-exposed animal. Arrowhead in **b** indicates a focally hypoperfused region within the cerebellum. Scale bar, 2 mm

**Fig. 2:**
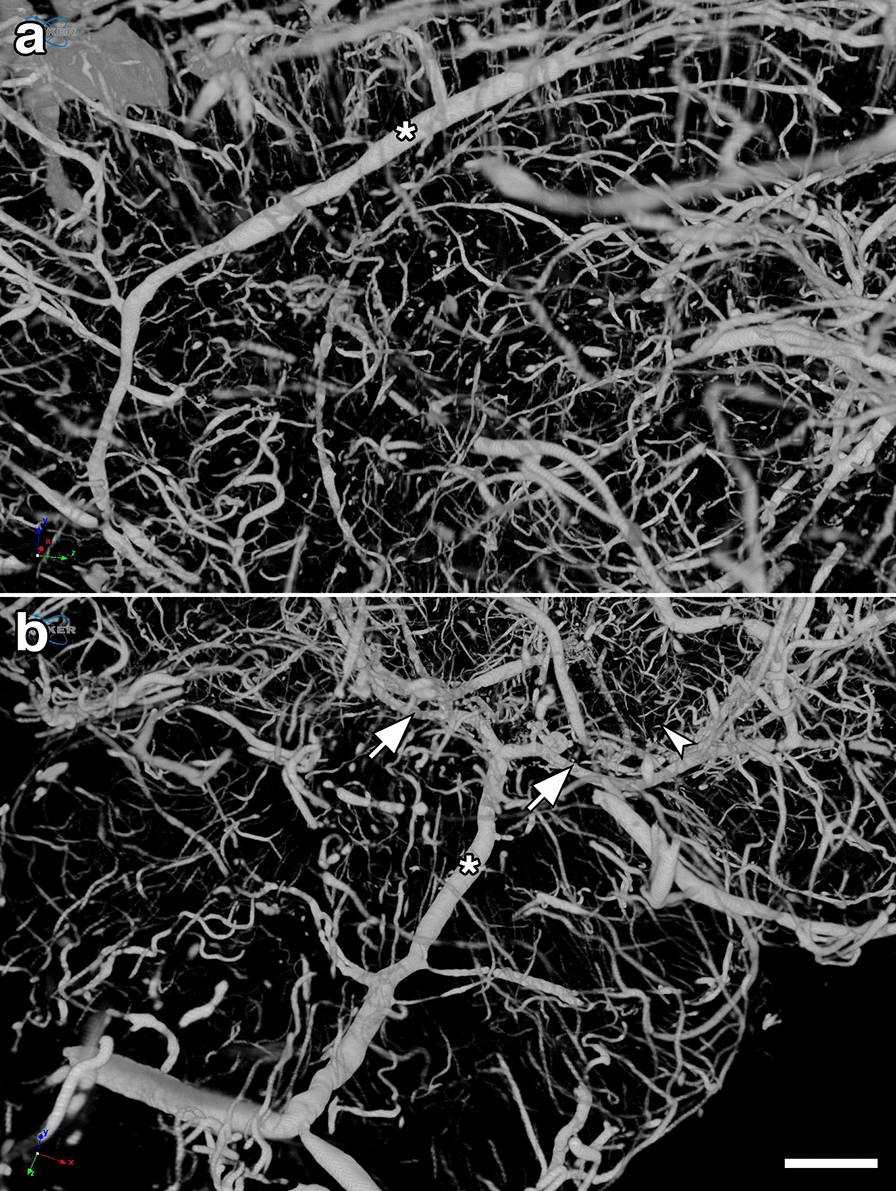
High resolution images of micro-CT scanning of the rat cerebral vasculature. **a** lateral view of the left middle cerebral artery (MCA, *) and (**b**), posterior view of the basilar (*) and the posterior cerebral arteries (arrows) from a control rat, 13 months post-sham exposure. Note visualization of large, medium and small vessels including what are likely precapillary arterioles (arrowhead). Scale bar, 1 mm

**Fig. 3:**
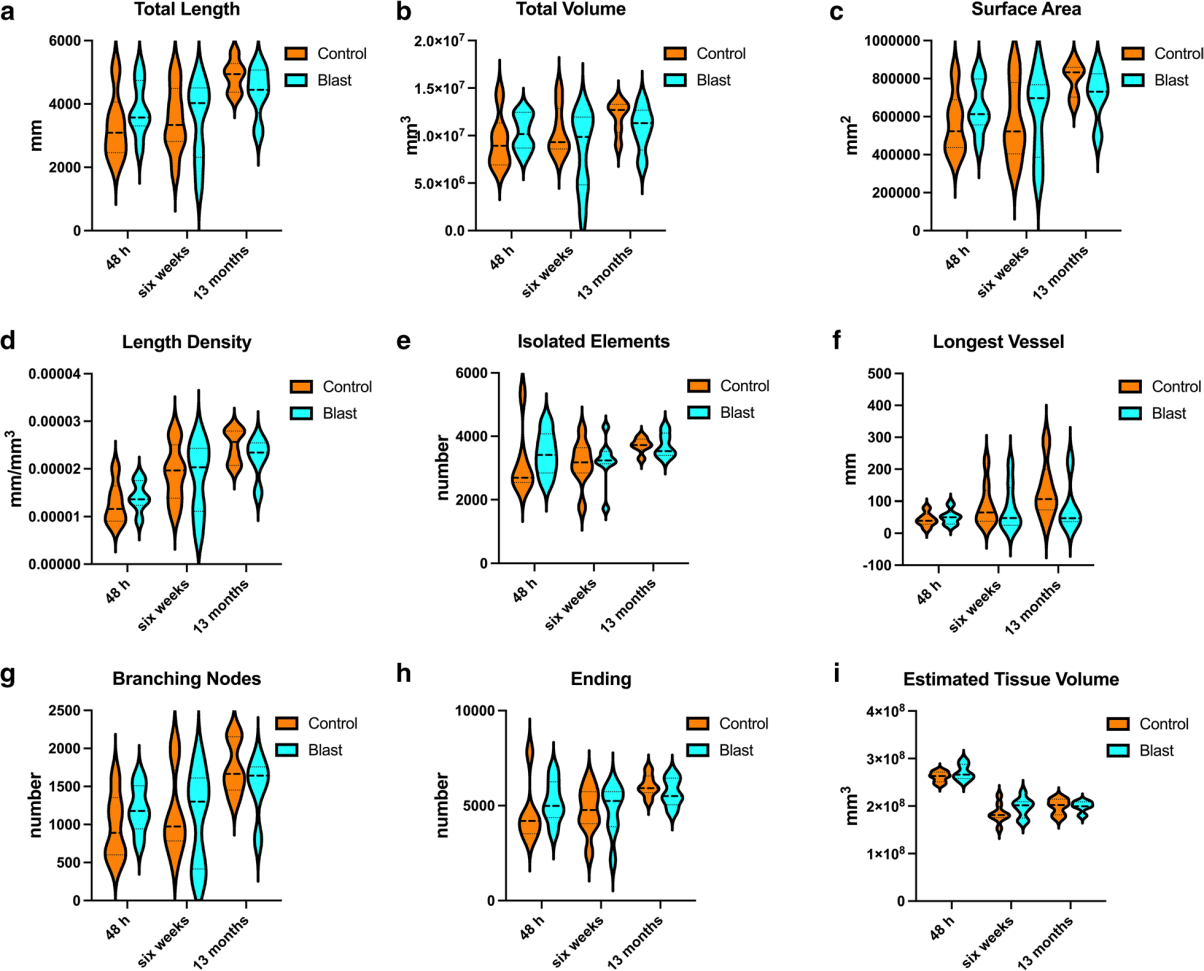
Quantification of X-ray high-resolution micro-CT scanning. Parameters were determined using the software Vesselucida Explorer (v2018.1.1, MBF Bioscience LLC, Williston, VT, USA). Shown is total vascular length **a**, volume **b**, surface area **c**, length density **d**, the number of isolated vessels **e**, the longest vessel **f**, the number of branching nodes **g**, ending vessels **h** and the estimated total tissue volume **i**. Data is shown as violin plots. There were no statistically significant differences between the blast-exposed and sham-exposed (control) cohorts at any of the times studied post-exposure

The evolution of the blast-induced chronic cerebral vascular pathology was investigated by X-ray high-resolution micro-CT scanning of brains of blast-exposed rats at 48 h, 6 weeks and 13 months post-blast exposure following perfusion with the Brite Vu contrast agent. Figure 1 shows representative maximum intensity projection (MIP) images of the volume-rendered brain vasculature of control and blast-exposed animals at the three time points. In Fig. 2, magnified high resolution micro-CT images are shown which reveal that in addition to large and medium sized arteries, smaller vessels including precapillary arterioles and possibly venules could be imaged. Quantitative analyses of the total cerebral vasculature (Fig. 3) did not show significant differences in major parameters, including length, surface area and volume, of the cerebral vasculature between blast-exposed and controls at any of the time points.

**Fig. 4:**
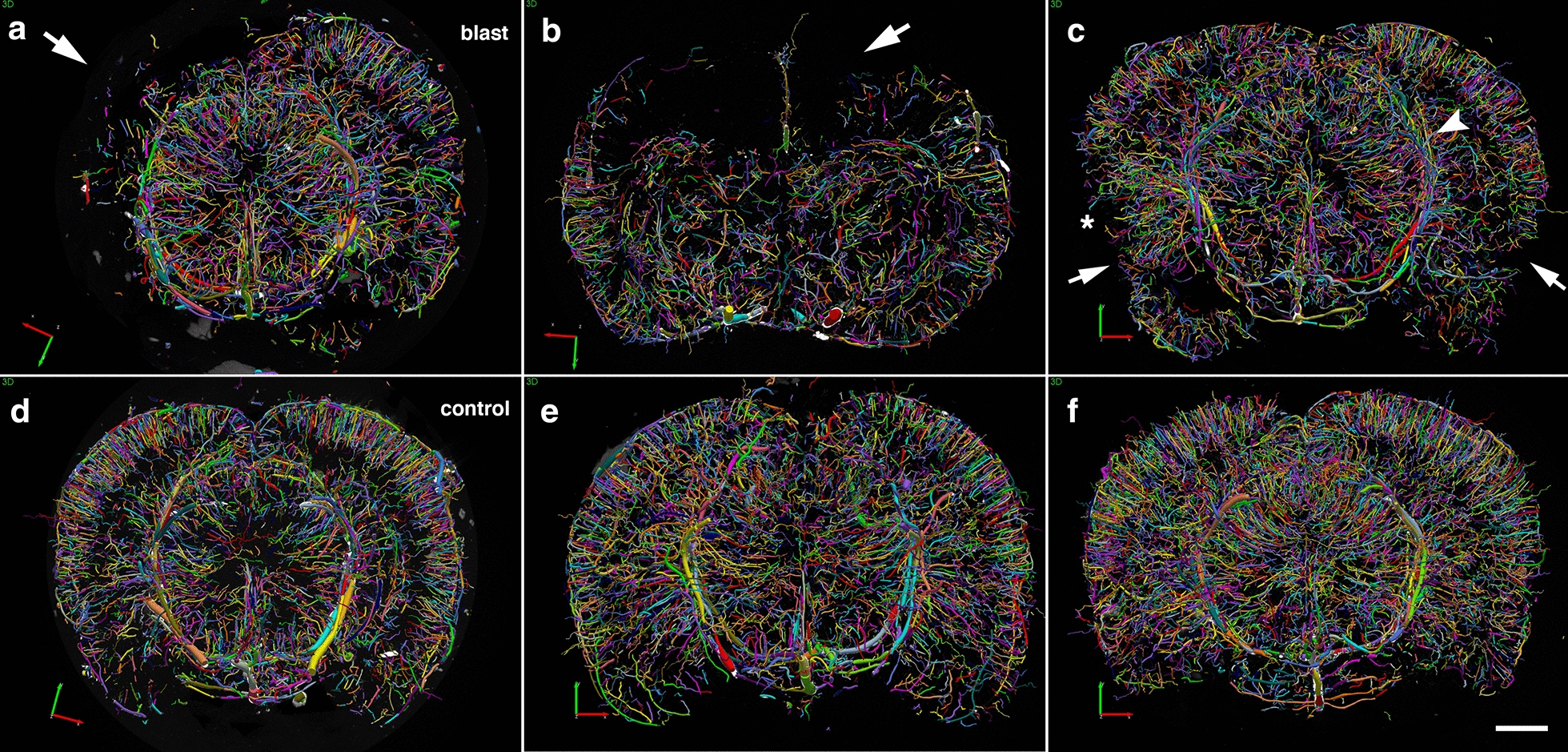
Acute, subacute and chronic vascular hypoperfusion and disorganization in the brains of blast-exposed rats. Brain micro-CT coronal optical sections (3.75 mm thickness) of blast-exposed and control rats perfused with the Brite-Vu-contrasting agent and reconstructed with the Vesselucida 360 software. **a** 48 h post-blast; **b** 6 weeks post-blast; **c** 13 months post-blast; **d**–**f** 48 h-, 6 weeks- and 13 months sham controls, respectively. Optical sections **a** and **d** correspond approximately to coordinates interaural 4.06–7.56 mm; **b** and **e** to interaural 6.36–9.86 mm; **c** and **f** to interaural 4.06–7.56 mm [97]. Note hypoperfused areas in the brains of blast-exposed rats relative to controls. The arrow in **a** indicates hypoperfusion of the entire left hemispheric cortical and hippocampal areas. Affected areas in **b** include the somatosensory, visual, motor and retrosplenial cortical regions whose circulation is derived from branches of the middle cerebral artery (MCA). In **c** blast-induced cerebral disorganization includes focal tears across the amygdala and adjacent cortical regions (arrows), attenuation of arteries involved in posterior circulation (arrowhead) and hypoperfusion of cortical areas (*). Scale bar, 1.5 mm

Nevertheless, post-blast imaging analyses of regional optical coronal sections (3.75-mm thickness) revealed stochastic vascular alterations in blast-exposed animals with attenuation, occlusion or disruption of the regular radial patterns visible in the brains of control animals. For example, Fig. 4a shows a rat brain from the 48 h post-blast cohort with vascular alterations in the hippocampal and posterior cortical regions of the left cerebral hemisphere, most likely the result of occlusion or attenuation of the posterior branches of the MCA as well as collateral branches of the posterior cerebral artery (PCA) and the anterior choroidal artery (AChA).

**Fig. 5:**
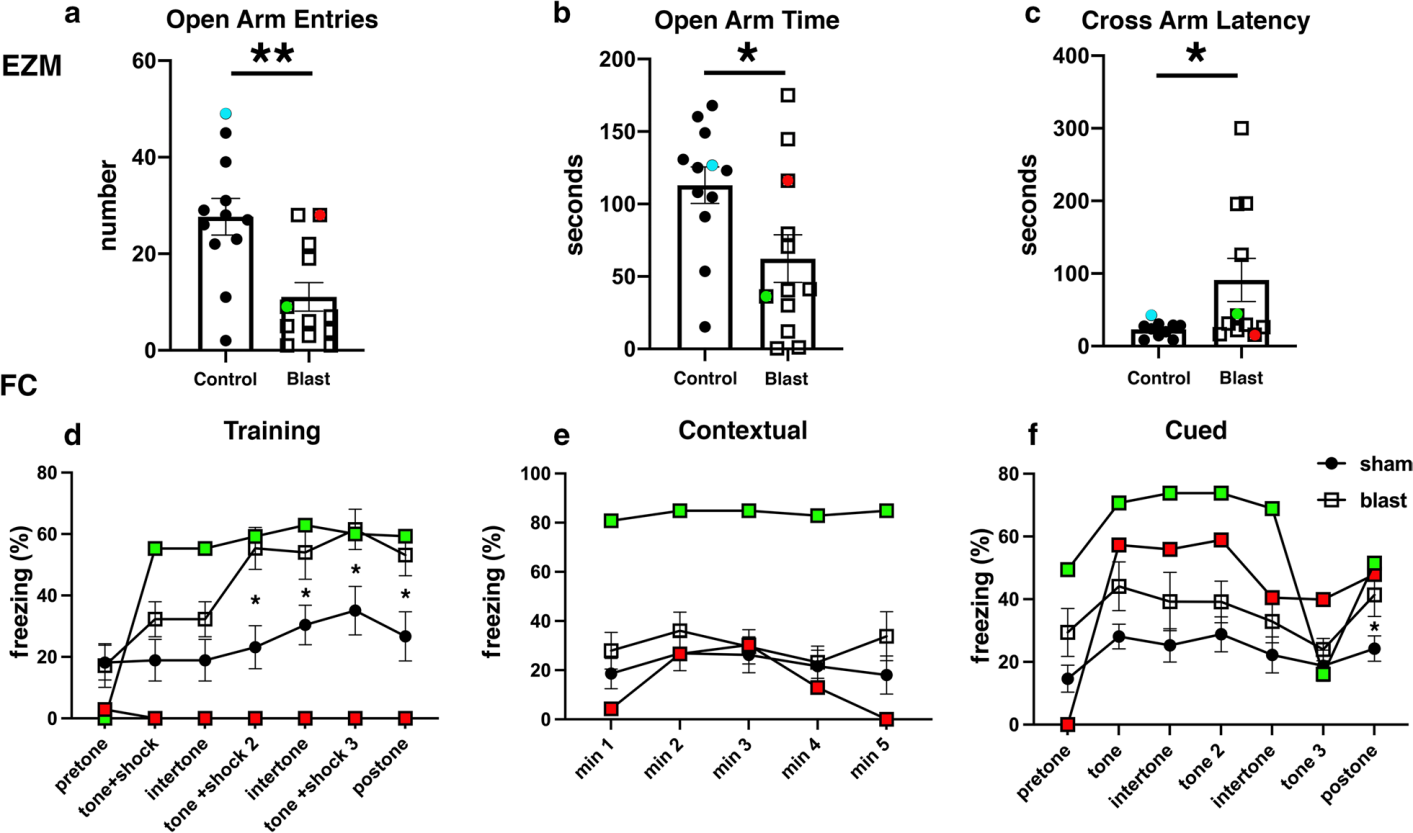
Correlation of behavioral traits with blast-induced cerebrovascular lesions in rats exposed to repetitive low-level blast at 13 months post-exposure. Elevated zero maze (EZM, **a**–**c**) and fear conditioning (FC, **d**–**f**) testing was performed as part of the studies described in ref [99]. EZM testing was performed at 40 weeks post-blast exposure. Shown for EZM are **a** open arm entries, **b** time spent in the open arms and **c** latency to cross between two open arms. Labeled in color are results from selected rats whose cerebral vasculature is illustrated in Figs. 4 (Fig. 4c, blast-exposed, red square; Fig. 4f, control, aqua circle) and 8 (Fig. 8a, b, blast-exposed, green square). FC testing was performed 42 weeks post-blast. Results are shown for the **d** training phase, **e** contextual fear memory testing (assessed 24 h after training) and **f** cued fear memory testing (assessed another 24 h later). Results of selected blast-exposed rats (Figs. 4c, red and 8a, b, green), are compared to the mean ± standard error measurements of blast and control cohorts determined previously [99]. Statistically significant differences between blast-exposed and control cohorts are indicated (*p < 0.05; **p < 0.01, unpaired *t* tests) [99]

At 6 weeks and 13 months post-blast, major alterations and disorganization of the cerebral vasculature were also observed. At 6 weeks post-blast, half of the blast-exposed rats (3/6) exhibited vascular alterations. Figure 4b shows attenuation of the vasculature irrigating the cingulate, motor, somatosensory, and retrosplenial cortex most likely involving the rostral branches of the MCA. At 13 months post-blast, cerebral vascular alterations were clearly observed in all blast-exposed animals (4/4) and none of the sham control rats (5/5) (Fig. 4c). In these animals, tissue tears, induced by the initial blast wave injury were clearly visible [39] (Fig. 4c). For example, in Fig. 4c lesions extend through the perirhinal or piriform cortical regions as well as the external capsule and amygdala and are similar to those described in previous blast-exposed cohorts [38, 39]. In the animal shown in Fig. 4c, blast-induced injuries resulted in bilateral amygdalar lesions. Interestingly, this animal which had been previously characterized behaviorally [95] was a relative outlier among blast-exposed animals in an elevated zero maze (EZM; Fig. 5a-c) making more open arm entries (Fig. 5a) as well as spending more time in the open arms (Fig. 5b) and exhibing a short cross arm latency (Fig. 5c). In a fear conditioning task (Fig. 5d–f), this animal showed no freezing responses during the training session (Fig. 5d) but exhibited relatively normal freezing during the contextual (Fig. 5e) and cued fear testing (Fig. 5f). In Fig. 5, behavioral data from the control animal illustrated in Fig. 4f is shown for comparison.

### Perivascular astrocytic degeneration, loss, and mislocalization

**Fig. 6:**
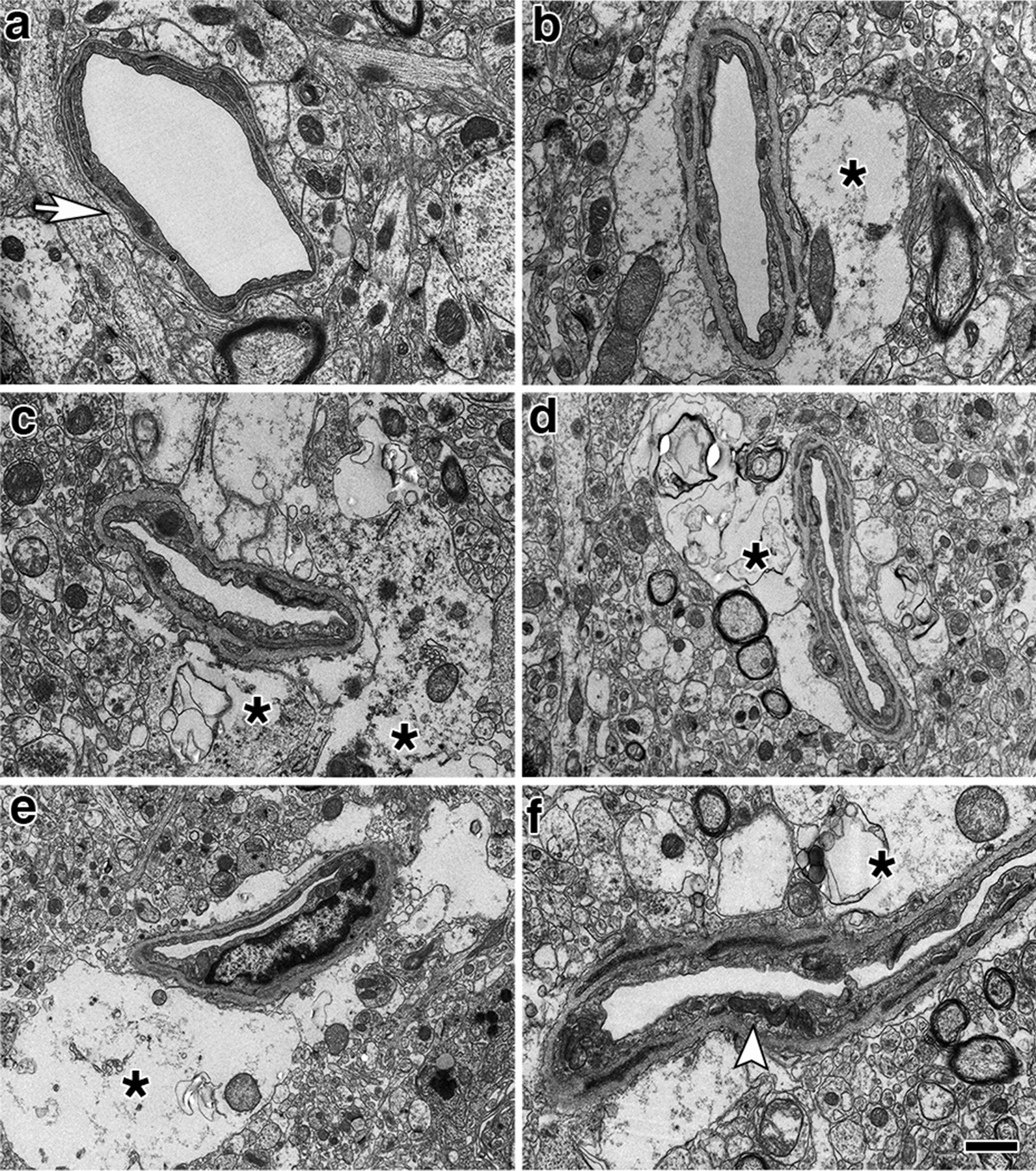
Astrocytic degeneration in the rat brain 13 months post-blast exposure. Electron micrographs of frontal cortical arterioles from control **a** and blast-exposed **b**–**f** rats. (*) indicates swollen astrocytic feet at different stages of degeneration. Arrow in **a** indicates a normal astrocytic endfoot. Arrow head in **f** shows an elongated mitochondria in an endothelial cell. Scale bar, 1 µm **a**–**c**, **f**; 1.5 µm **d**; 2 µm **e**

**Fig. 7:**
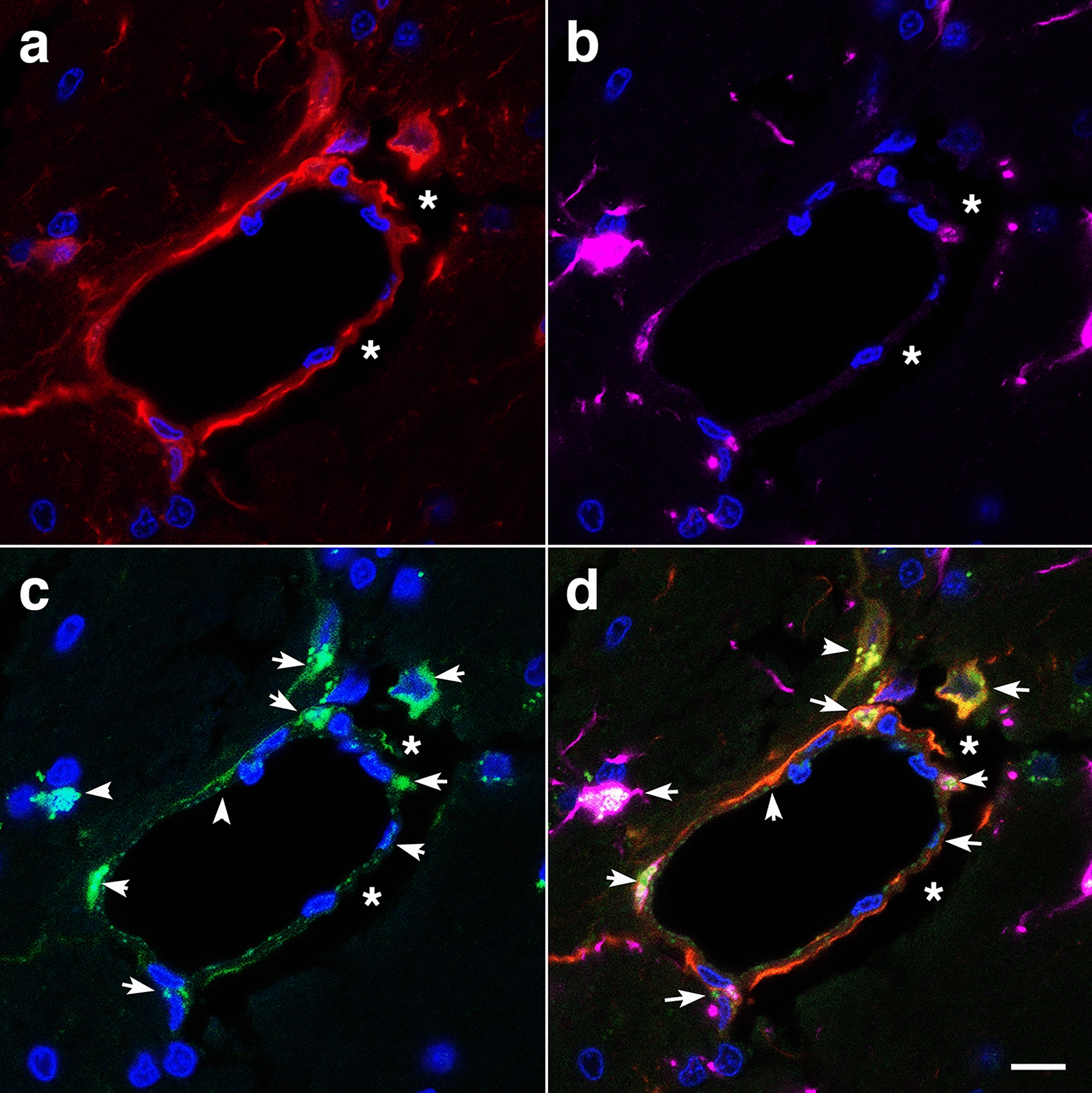
Perivascular apoptosis in a vessel with enlarged paravascular spaces. A hypothalamic vessel showing perivascular astrocytes (**a**, GFAP, red) and microglia (**b**, Iba1, magenta) analyzed by TUNEL staining (**c**, green), merged **d**. DAPI (blue). (*) denotes the enlarged paravascular spaces. TUNEL staining of perivascular astrocytes and of astrocyte-associated microglia is indicated by arrows. Scale bar, 25 µm **a**–**d**

**Fig. 8:**
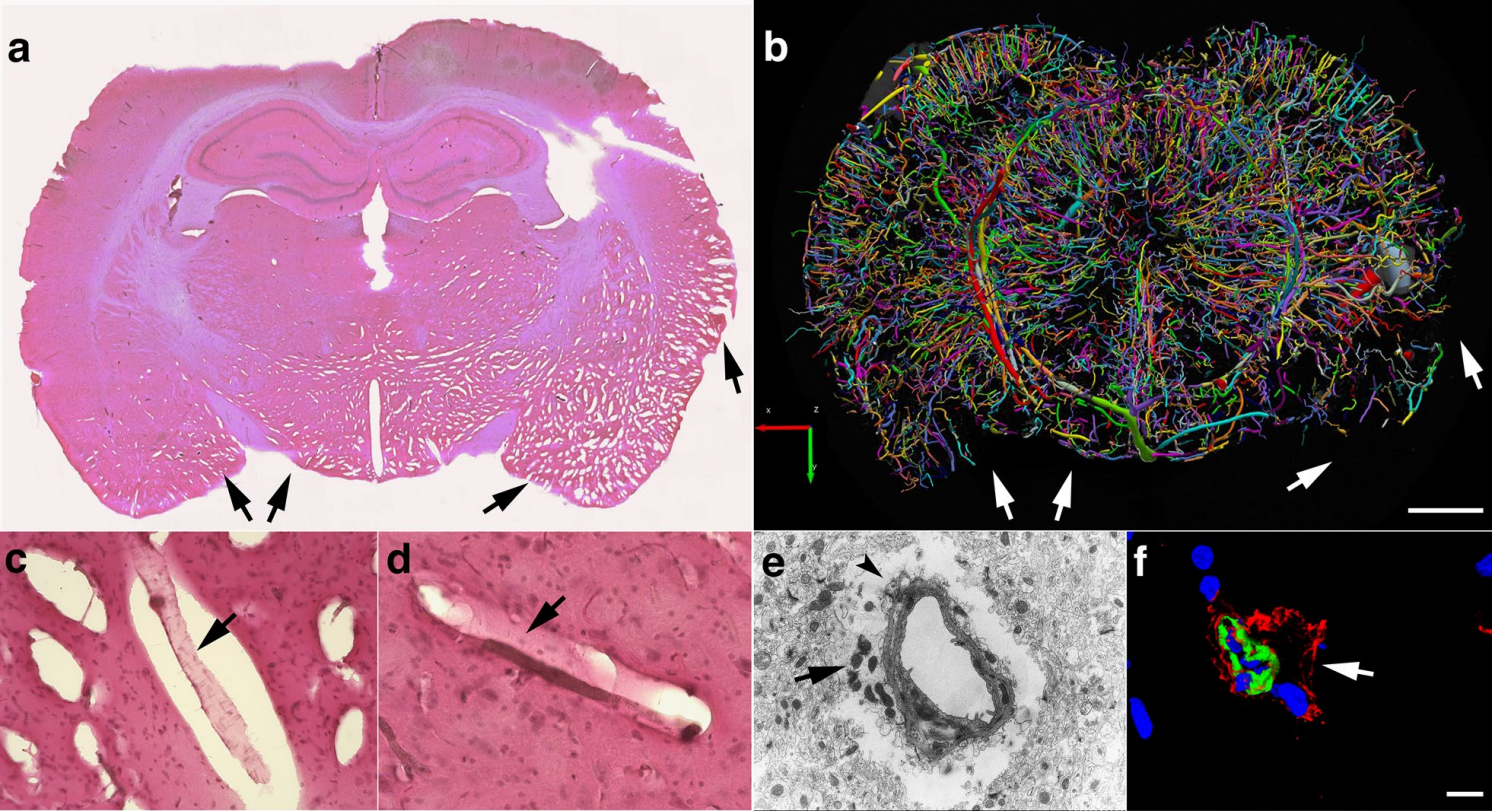
Chronic enlargement of paravascular spaces in the brain at 13 months post-blast exposure. Coronal sections from a blast-exposed animal were stained with hematoxylin and eosin **a**, **c**, **d**. The general pathology showing enlarged paravascular spaces is shown (arrows in **a**). In this animal, the histopathology is mostly concentrated in one hemisphere and involves the amygdala, insula, piriform cortex, ventral thalamus and hypothalamus. In the contralateral hemisphere, pathological changes are limited to the piriform cortex and hypothalamus. Panel **b** shows the reconstructed cerebral vasculature from a 3.75 mm micro-CT optical section that includes the region shown in **a**. The vascular reconstruction **b** mirrors the enlarged paravascular spaces seen in panel **a** where the hypothalamus, ventral thalamus and, most strikingly, the amygdala and piriform cortex show reduced perfused vessels (arrows) and enlarged paravascular spaces. The lack of vascular tracing (arrows in **a**, **b**) suggests hypoperfusion of the corresponding areas and could be a consequence of perivascular astrocytic degeneration. Panel **c** shows a detached artery (arrow) within an enlarged paravascular space in the ventromedial hypothalamus. Panel **d** shows a constricted artery (arrow) in the ventral posteromedial nucleus from which the adventia has been detached. Panel **e** shows an electron micrograph of a cortical arteriole barely attached to the parenchyma through a few degenerating astrocytic endfeet (arrow). Arrowhead in **e** shows enlarged paravascular space. Panel **f** shows immunohistochemical analyses of a hypothalamic arteriole with a disrupted smooth muscle layer (α-SMA^+^, green) and detached adventitia (col IV^+^, red); DAPI, blue. Scale bars, 2 mm **a**, **b**; 40 µm **c**, **d**; 1.5 µm **e**; 15 µm **f**

We previously described the blast-induced degeneration of astrocytes surrounding the cerebral vasculature as already ongoing at 6 weeks post-exposure [40]. To evaluate the astroglial coverage of the brain vasculature at 13 months post-blast, sections of control and blast-exposed animals were analyzed first by electron microscopy (Fig. 6). Electron micrographs showed long-term blast-induced effects on perivascular astrocytes including swelling and degeneration of astrocytic endfeet in the brain cortical vasculature. The processes of astrocytic endfeet in an advanced degenerating phase were devoid of cellular organelles (Fig. 6). Some small vessels and arterioles had enlarged paravascular spaces that reflected the loss of perivascular astrocytes bridging the parenchymal neuropil and the vessel. Apoptotic perivascular astrocytes and microglia were detected by Tunel staining associated with vasculature with enlarged paravascular spaces (Fig. 7). These observations are in agreement with the development of an unusual vascular pathology observed in two additional animals from the 13 months post-blast cohort (Fig. 8). This pathology, characterized mainly by the presence of vascular segments within enlarged paravascular spaces (Fig. 8), often affected mainly one cerebral hemisphere. The brain regions affected generally included the cerebellum, hypothalamus, thalamus, neocortex, hippocampus, basal ganglia, piriform cortex and amygdala (Fig. 8a). Vascular reconstructions of micro CT optical sections showed that brain regions with enlarged paravascular spaces also showed drastic reductions of micro-CT-traced vessels. As an example, Fig. 8a, b shows that enlarged paravascular spaces in the hypothalamus, ventral thalamus and, most strikingly, the amygdala and piriform cortex have a clear reduction of micro-CT-traced vessels. The lack of vascular tracing within regions with enlarged paravascular spaces is indicative of vascular hypoperfusion or attenuation and could be a consequence of lack of vascular tone regulation due to perivascular astrocytic degeneration. In behavioral testing this animal exhibited an anxiety phenotype in an EZM that was similar to other blast- exposed animals. (Fig. 5a–c). In fear conditioning, it exhibited a training curve (Fig. 5d) relatively similar to other blast-exposed animals but showed increased freezing in contextual (Fig. 5e) and cued (Fig. 5f) fear testing. Histochemical, immunohistochemical and electron microscopic analyses showed that the vasculature enclosed within the enlarged paravascular spaces involved mainly arterial vessels (Fig. 8c–f) and in some of these vessels the adventitial layer could be found detached (Fig. 8d, f).

**Fig. 9:**
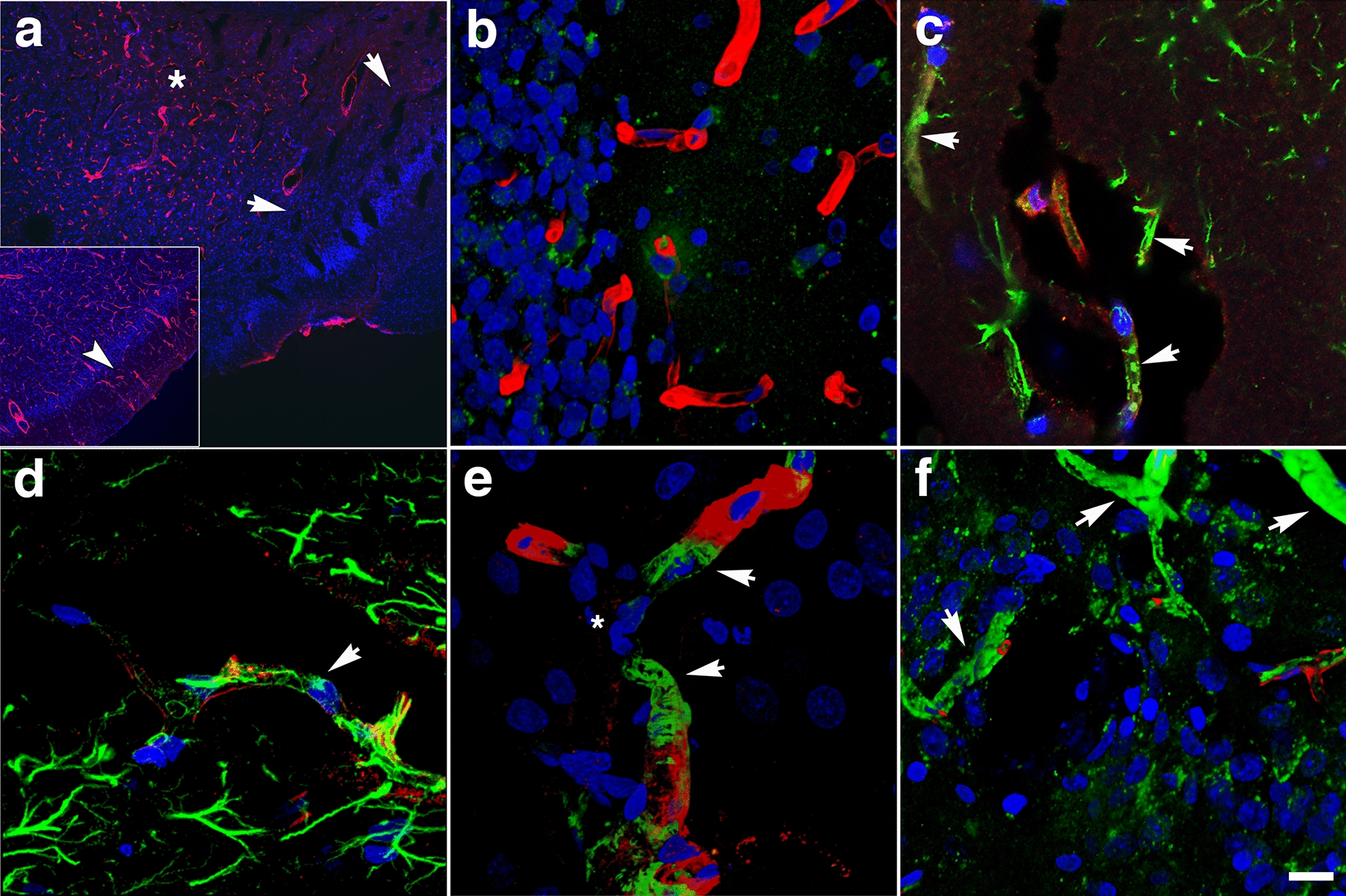
Loss of vascular collagen type IV in brain regions with enlarged paravascular spaces in rats at 13 months post-blast exposure. Coronal brain sections of a blast-exposed rat were treated with pepsin to unmask the collagen type IV epitopes and stained with rabbit polyclonal antibodies against collagen type IV (red). **a** Lack of collagen type IV immunostaining (red) in the piriform cortical region exhibiting large paravascular spaces (arrows). Note the normal collagen IV staining (red) in the adjacent areas (asterisk). The inset in **a** corresponds to a section from a control animal showing normal collagen type IV staining (arrow) of the vasculature in the piriform cortex and neighboring regions. **b** Normal vascular collagen type IV staining of the hippocampal vasculature in the same blast-exposed animal (coll IV, red; GFAP, green). **c**, **d** Loss of collagen IV immunostaining in small vessels associated with hypothalamic enlarged paravascular spaces. GFAP^+^ perivascular astrocytic endfeet remained attached (GFAP, green, arrows). **e** Loss of collagen type IV in the arterial adventitia in regions with enlarged paravascular spaces (α-SMA, green). Arrows in **e** show arterial regions devoid of collagen IV (red) around a vascular stricture (*). Also note the loss of smooth muscle (α-SMA^+^, green) around the stricture. **f** Loss of adventitial collagen IV (arrows) in hippocampal arterioles (coll IV, red; α-SMA, green). DAPI, blue. Scale bar, 140 µm **a**; 20 µm for **b**–**f**

**Fig. 10:**
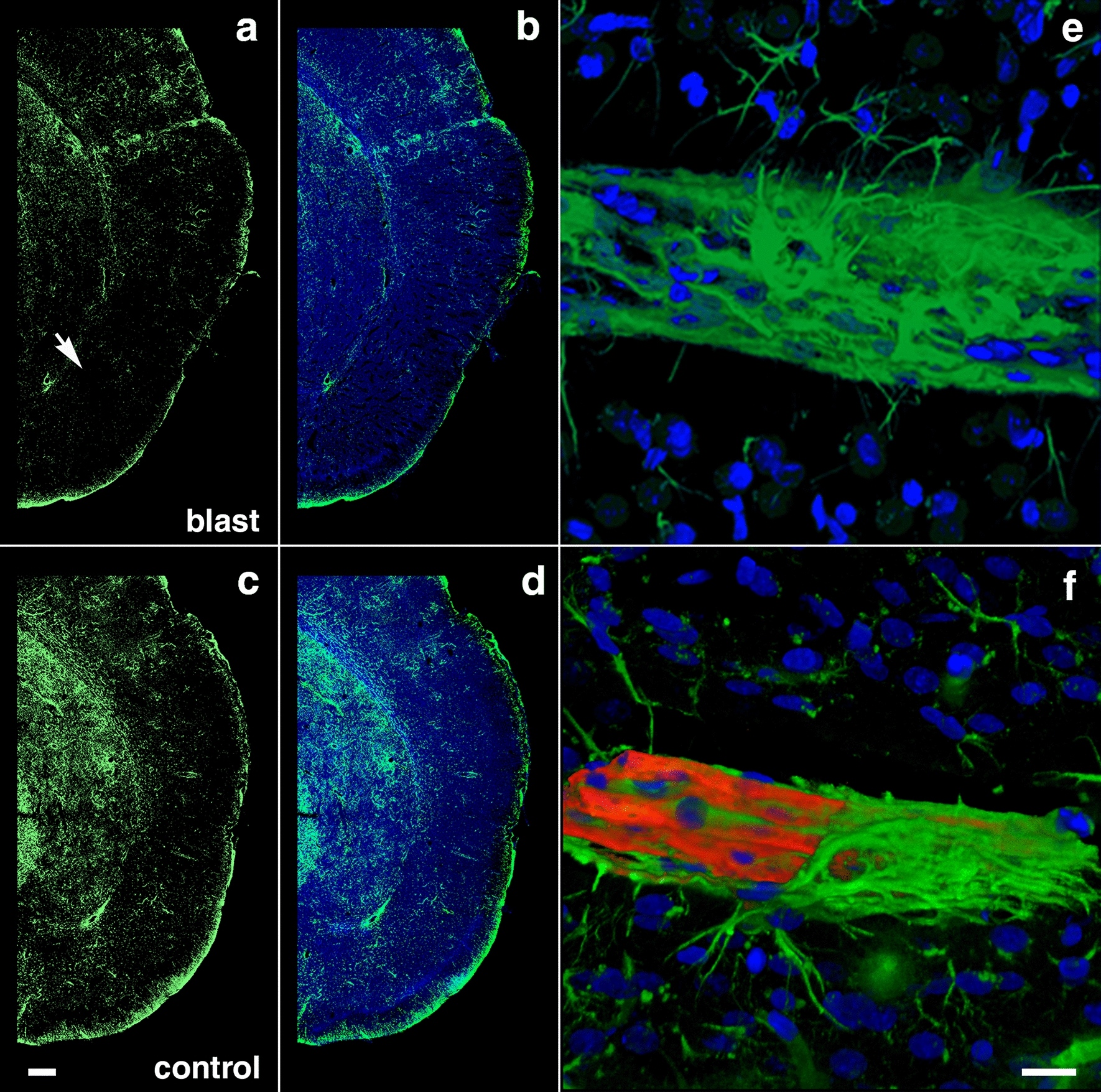
Reduced GFAP immunoreactivity in brain regions with enlarged paravascular spaces of blast-exposed rats at 13 months post-blast exposure. Coronal brain sections from blast-exposed **a**, **b** and control **c**, **d** rats were immunostained with antibodies against GFAP (green) and counterstained with DAPI (blue in merged images, **b**, **d**). GFAP immunoreactivity was greatly reduced in the thalamus and hypothalamus of blast-exposed animals harboring enlarged paravascular spaces (arrow in **a**). **e**, **f** Abnormally high perivascular astrocytic density in a few large vessels in the entorhinal cortex of blast-exposed rats. Section in **e** was single-stained with GFAP (green) and **f** was double-stained for GFAP (green) and collagen type IV without protease pre-treatment (red,). DAPI (blue). Panel **f** also shows GFAP^+^ (green) cells under the adventitial collagen type IV (red). Scale bars, 320 µm **a**, **b**, **d**, **e**; 16 µm **c**, **f**

As previously reported [3, 38, 40], some of the arterioles presented increased tortuosity and structural smooth muscle alterations as strictures and tortuosity could result in loss of smooth muscle (Fig. 9e). GFAP and collagen type IV immunostaining revealed collagen IV degradation associated with loss of perivascular astrocytes in the affected vasculature (Figs. 7, 9, 10). Compared to unaffected brain regions, loss of vascular collagen IV immunostaining was seen in areas with enlarged paravascular spaces (Fig. 9a–f). Although perivascular astrocytes were lost in the affected vasculature and immediate surrounding parenchyma, remains of GFAP^+^ astrocytic feet could be seen remaining attached in some vessels (Fig. 9c).

### Remodeling of the adventitial ECM

**Fig. 11:**
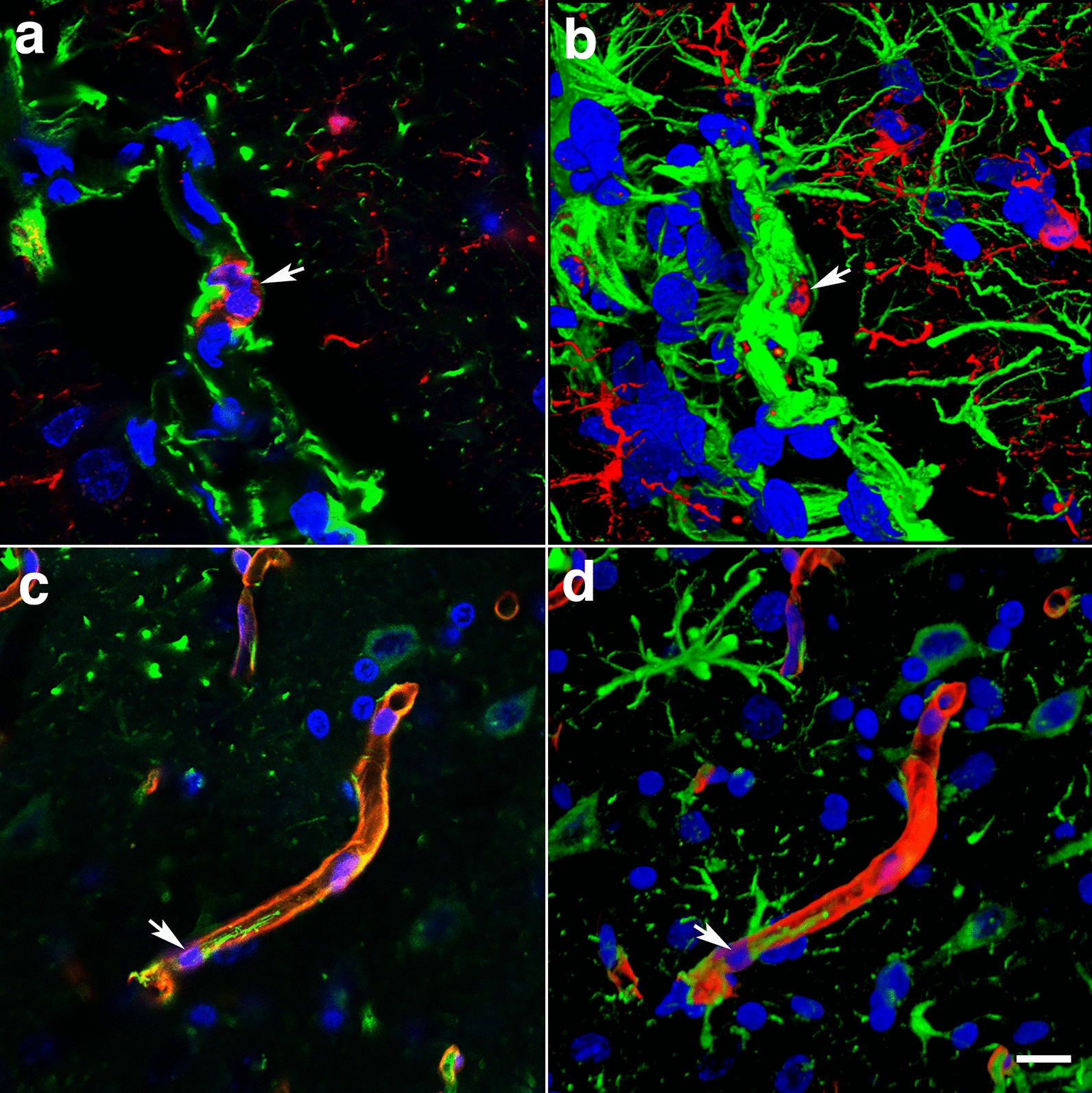
Microglia/macrophage and astrocytic cells in the vascular lumen. **a** Confocal optical section (0.56 µm) showing an Iba1^+^ microglia/macrophage (arrow) inside the vascular lumen (Iba 1, red; GFAP, green). Panel **c** shows an astrocytic cell (arrow) and its process inside the lumen of a small vessel (GFAP, green; coll IV, red). Panels **b** and **d** correspond to three-dimensional stack reconstructions of the fields shown in panels **a** and **c**. DAPI, blue. Scale bar, 15 µm for **a**, **b**; 20 µm for **c**, **d**

In addition to the loss of perivascular astrocytes, some vessels showed mislocalization of astrocytes. We previously reported the presence of intravascular GFAP^+^ astrocytic processes in the cerebral vasculature of animals at 6 weeks post-blast exposure [40]. At 13 months post-blast, confocal microscopy identified astrocytes under the adventitial ECM scaffold (Fig. 10f) and in the lumen (Fig. 11c, d). This implies ECM remodeling by adventitial fibroblasts along with perivascular astrocyte repositioning within the vascular internal structural layers. The presence of intraluminal astrocytes or astrocytic processes may identify non-functional degenerating vasculature into which perivascular astrocytes and/or their processes could extend.

**Fig. 12:**
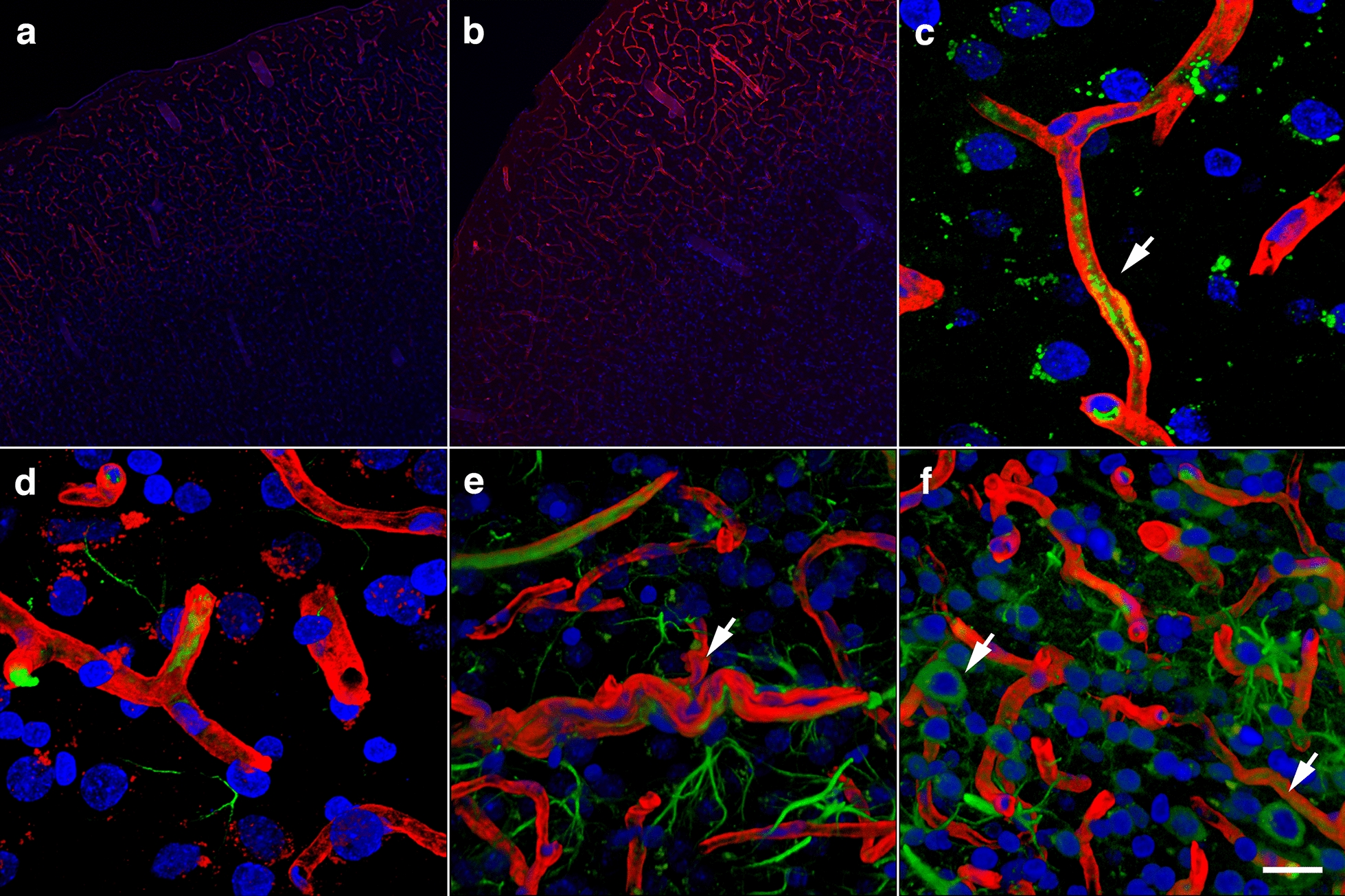
Chronic proteolysis of collagen type IV in the brains of blast-exposed rats. Unmasking collagen type IV epitopes in the vasculature of normal mature adult rodent brain for immunohistochemical detection requires protease pretreatment [37]. Shown are micrographs of collagen type IV-immunostained sections of brains of control and blast-exposed rats at 13 months post-blast exposure *without* protease treatment. **a**, **b**, Vasculature in the somatosensory cortex of control **a** and blast-exposed **b** rats immunostained for collagen type IV (red). Note the absence of collagen IV staining in the section from the control rat **a**. **c** A small vessel immunostained for collagen type IV (red) showing MMP-9 vescicles (green, arrow). **d**–**f** Collagen type IV (red) and GFAP (green) immunoreactivity of the somatosensory cortical vasculature of blast-exposed rats in the absence of protease pretreatment. Note the tortuosity of the vessel shown in **e** and the neuronal expression of GFAP (arrows) in this region **f**. DAPI, blue. Scale bar, 140 µm for **a**, **b**; 20 µm for **c**–**f**

Adventitial fibroblasts become involved in vascular remodeling via their regulation of ECM degradation mediated by MMPs and tissue inhibitors of metalloproteinases (TIMPs). The presence of vascular protease activity in rats at 10 months post-blast has previously been demonstrated by the immunostaining of the cerebral vasculature with collagen type IV antibodies without prior protease treatment [38]. Similarly, at 13 months post-blast, the cerebral vasculature was extensively stained with anti-collagen type IV antibodies in the absence of a protease treatment. In age-matched controls, collagen type IV vascular staining was very limited (Fig. 12a, b).

**Fig. 13:**
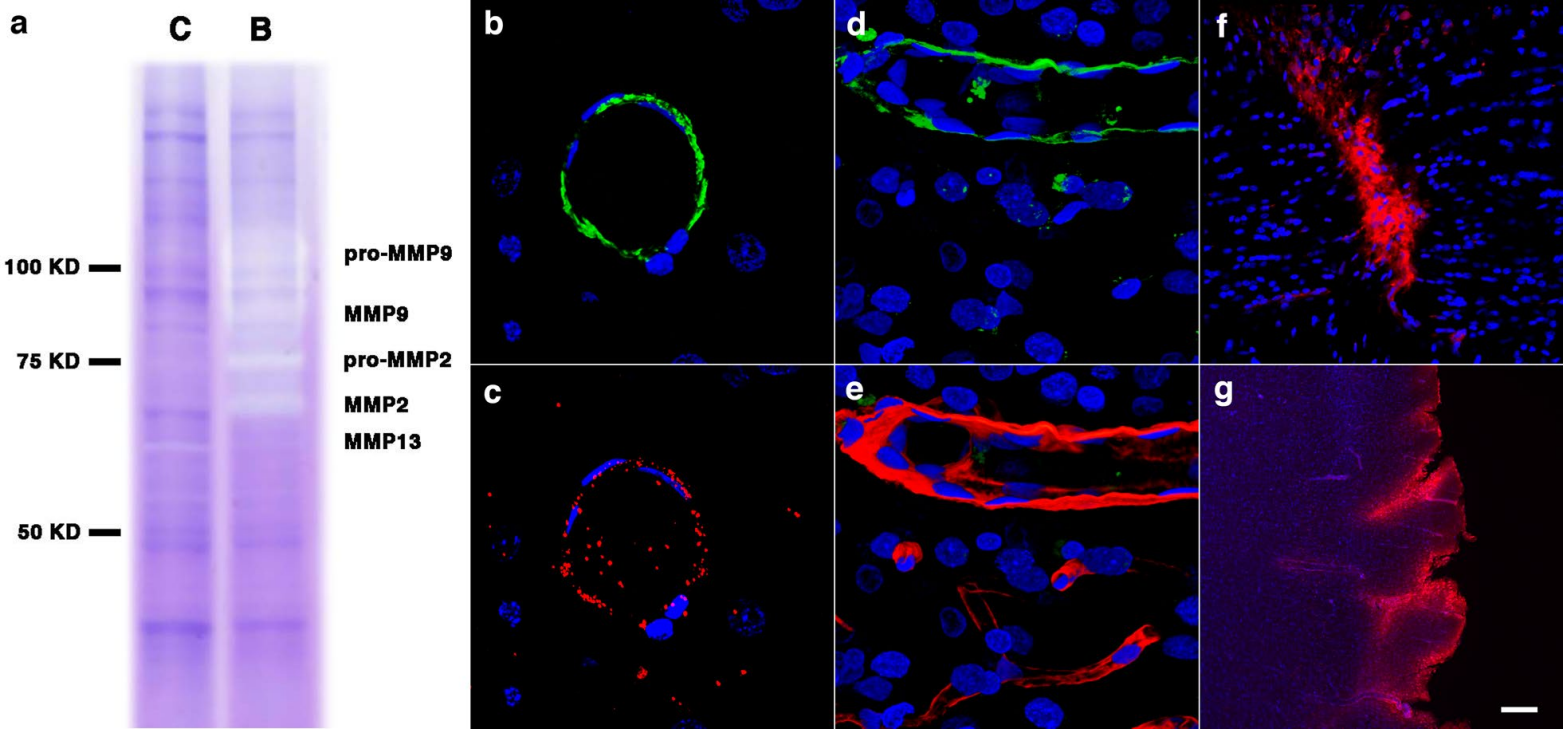
Metalloproteases in the brain of blast-exposed rats. Enriched brain vascular fractions were prepared from control and blast-exposed rats at 6 weeks post-exposure. Protein extracts (20 µg) from the enriched vascular fractions were analyzed by gelatin zymography. Panel **a** shows vascular upregulation of gelatinase activity associated with MMP-2, MMP-9 and their precursor proteins in the blast-exposed animal (B) as compared to control (C). Panels **b**, **c** show a brain arterial cross-section from a blast-exposed rat stained with antibodies against α-SMA (**b**, green) and MMP-9 (**c**, red). Panel** d** shows MMP-9 expression (green) in brain arterial smooth muscle of a blast-exposed rat at 13 months post-exposure. Panel **e** showns the same vessel in **d** stained with collagen IV (red). Brain sections of blast-exposed animals were stained with anti-rat IgG (red) and counterstained with DAPI (blue) **f**, **g**. Leakage of IgG could be seen in perivascular areas within the corpus callosum **f** or the entorhinal cortex **g**. Scale bars, 20 µm **b**, **c** 10 µm **d**, **e** 100 µm **f**; 300 µm **g**

**Fig. 14:**
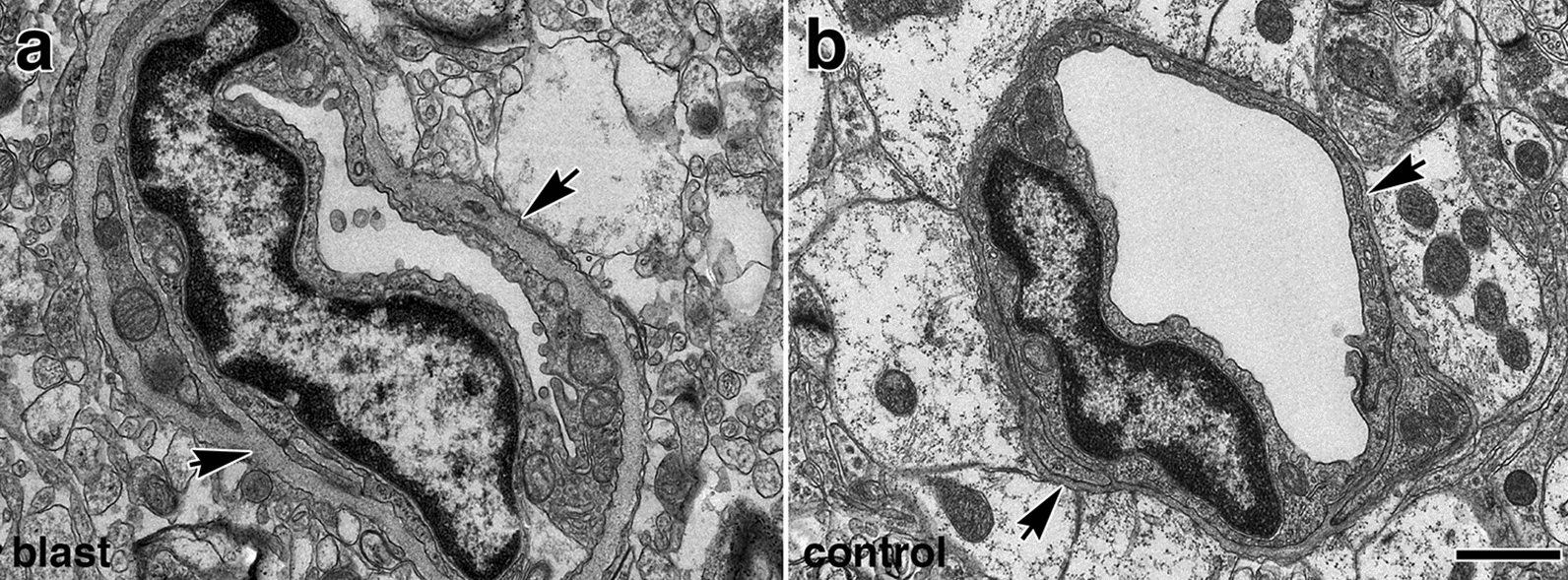
Blast-induced thickening of the basal lamina surrounding the brain vasculature at 13 months post-blast exposure. Electron micrographs of small vessels in the frontal cortex of blast-exposed **a** and control animal **b**. Note that in the vessel from the blast-exposed animal **a** compared to control **b**, there is enhanced thickness of the vascular basal lamina (arrows), a reduction of luminal space, swelling of the endothelial nucleus, and reduction of astrocytic endfeet contacts. The endothelial membrane in contact with the lumen is distorted and has an irregular "wavy" pattern. Also, note that in panel **a** the cytosol of the remaining astrocytes lack the ribosomal density present in the normal astrocytic cytoplasm of control vessels **b**. Scale bar, 1 µm

Increased levels of brain MMP-9, MMP-9 mRNA, and plasma MMP-9 have been observed acutely after a 130-kPa blast exposure in association with blood–brain barrier dysfunction [66]. Gelatin zymography of purified cerebral vascular fractions showed that levels of MMP-2 and MMP-9 could also be increased at 6 weeks post-blast exposure (Fig. 13a). At 13 months post-blast, the cerebral vasculature showed a reduction in collagen type IV immunoreactivity in some regions (Fig. 9), smooth muscle expression of MMP-9 (Fig. 13b–e) and thickening of the vascular laminae (Fig. 14). In addition, vascular IgG leakage was detected at this age suggesting that the peripheral circulation might be an additional source of proteases affecting the vasculature (Fig. 13f, g). These results illustrate the developing chronic vascular remodeling of the ECM in the vascular scaffold after blast exposure.

### Vascular-associated neuroinflammation

**Fig. 15:**
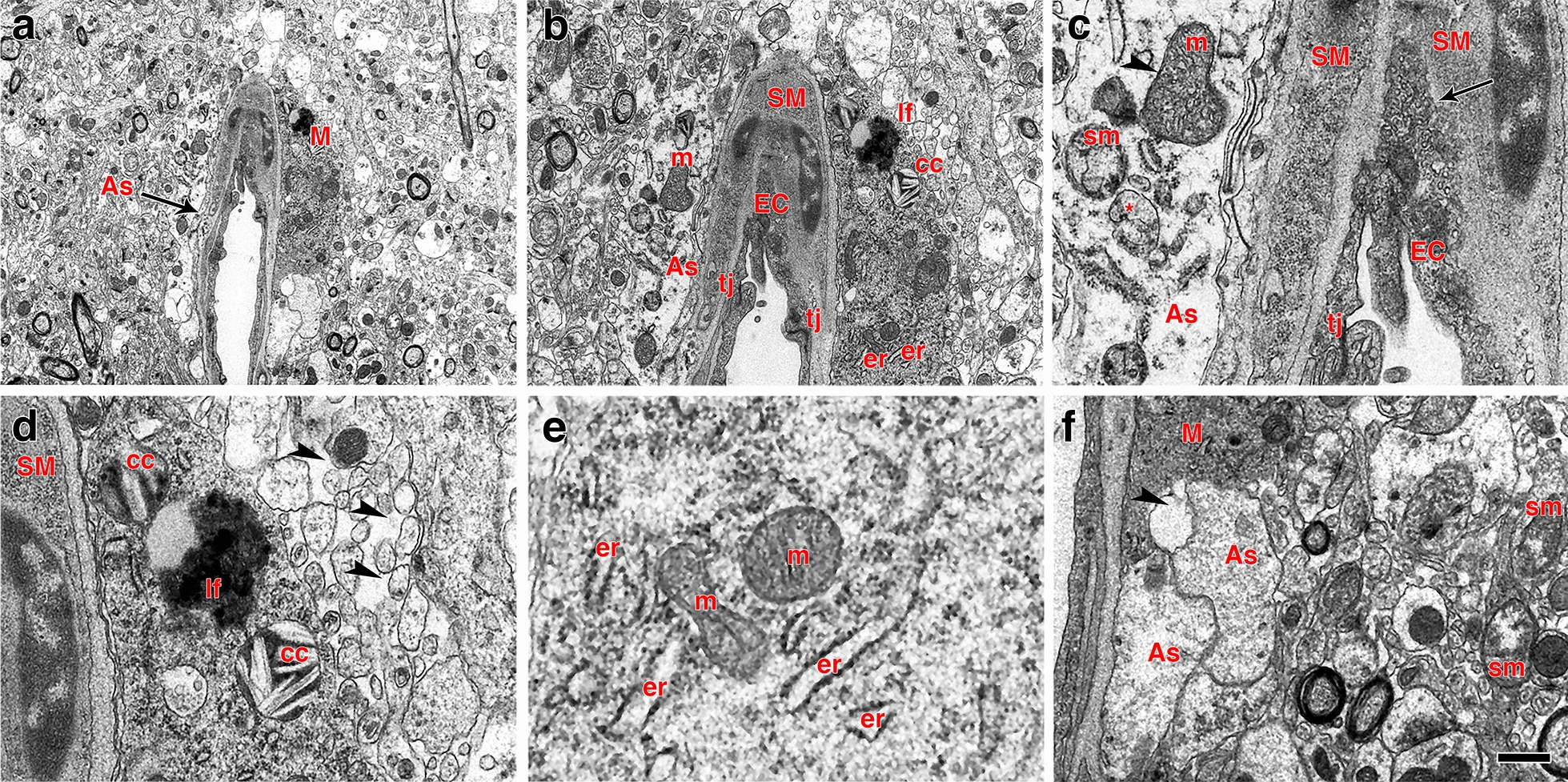
Perivascular microglia and vascular degeneration. **a**–**f** Electron micrographs of a perivascular microglial cell (M) adjoining a degenerating arteriole. Perivascular astrocytic feet (As, arrow) exhibit lack of ribosomes and altered mitochondria including swollen mitochondria (sm, panel **c**), mitochondria with disorganized cristae (arrowhead in **c**) and completely dystrophic mitochondria (* in **c**). The intimal endothelial cells (EC, panels **a**–**c**) are partially dislodged from the basal lamina with free processes protruding into the lumen. The abnormal endothelial cells exhibit a high density of small osmiophilic vescicles. Note the lack of definition of the basal lamina between endothelial and smooth muscle (SM) cells (arrow in **c**). The perivascular microglial cell **a**, **b**, **d**–**f** shows lysosomal cholesterol crystals (cc in panels **b** and **d**), a lipofuscin body (lf in panels **b** and **c**) and enlarged endoplasmic reticulum cisternae (er) characteristic of the “dark” microglial phenotype **e**. Arrowheads in panel **d** show abnormal enhanced interstitial spaces in the neuropil neighboring the perivascular microglial cell. Arrowhead in panel **f** indicates remnants of an astrocytic foot process next to a microglial process. m, mitochondria; tj, tight junction. Scale bars, 2.5 µm **a**; 1 µm **b**; 0.2 µm **c**; 0.4 µm **d**, **e**; 0.8 µm **f**

Microglial activation is a brain pathology hallmark that occurs in response to injury through damage-associated molecular patterns and removal of immunosuppressive signals. Electron microscopic observations at 13 months post-blast identified periarteriolar microglia with intracellular cholesterol crystals next to degenerating astrocytic endfeet (Fig. 15a, b, d).

**Fig. 16:**
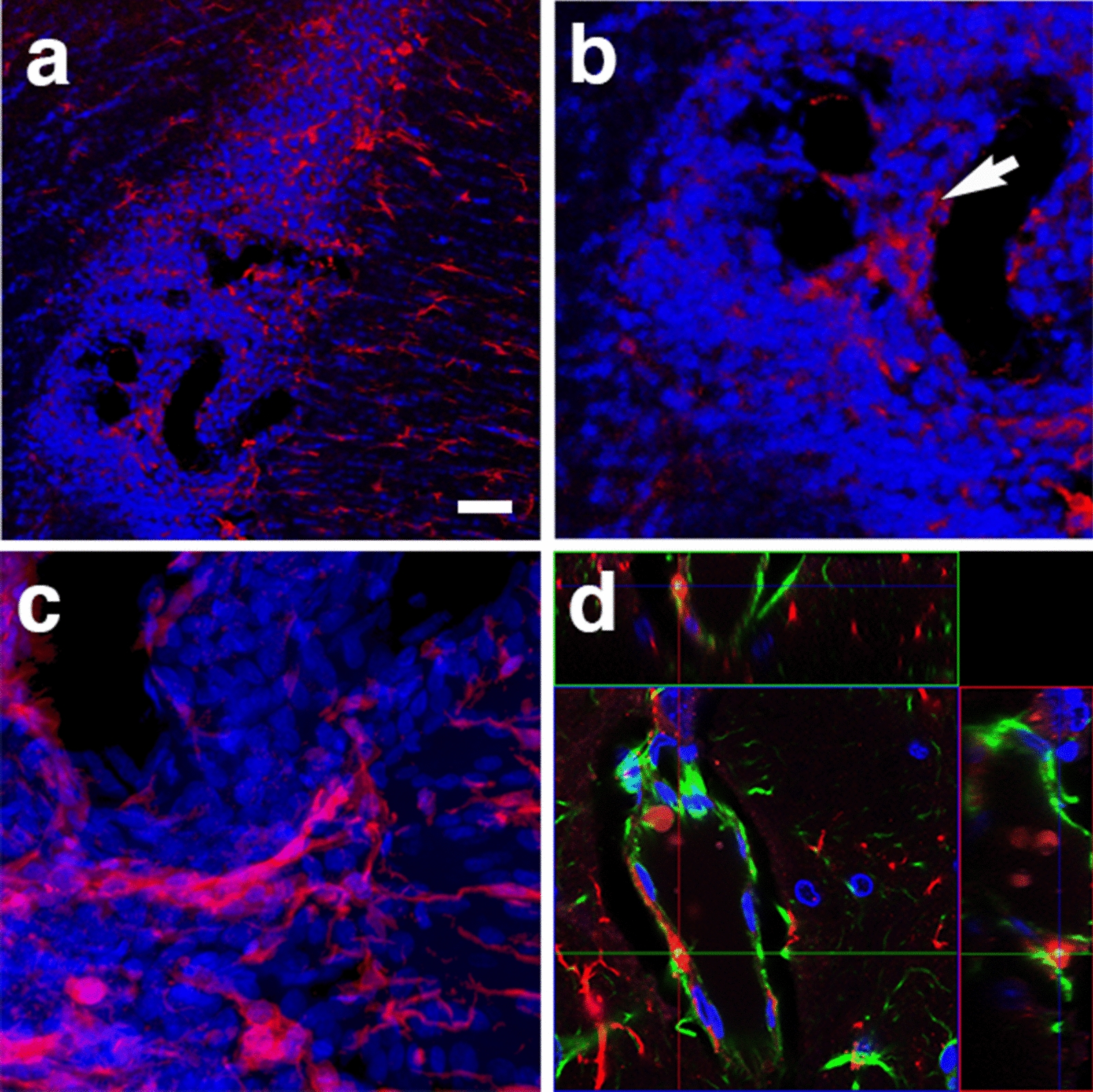
Perivascular microglia in the brain of blast-exposed animals. Panels **a**–**c** show clustered Iba 1^+^ microglia (red, arrow in **b**) with amoeboid morphology in the perivascular area of a tortuous large vessel (pericallosal artery) in the corpus callosum of a rat brain at 13 months post-blast exposure. Panel **d** shows an orthogonal projection of a section through a thalamic artery stained for perivascular astrocytes (GFAP^+^, green) and microglia (Iba1^+^, red). Co-localization of astrocytic GFAP^+^ material within a perivascular microglial cell, suggests phagocytic ingestion of perivascular astrocytes by microglia. Scale bar, 100 µm **a**; 40 µm **b**; 20 µm **c**, **d**

**Fig. 17:**
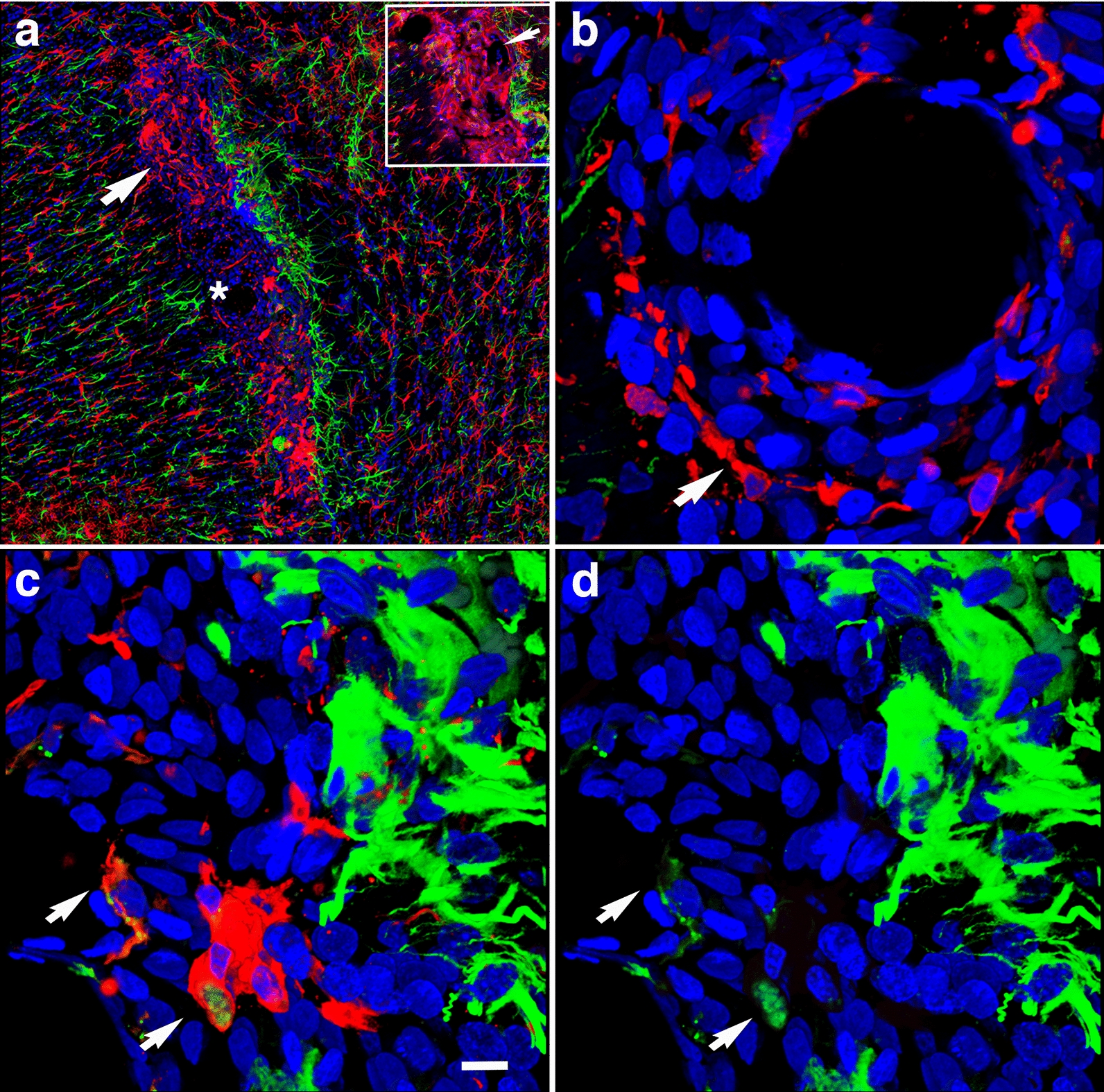
Perivascular inflammation in the brain of a blast-exposed animal. Panel **a** shows a brain region with patches of clustered activated microglia (Iba1^+^, red) next to a vascularized region in between the deep cerebral white matter, choroid plexus and hippocampal fimbria. Insert in **a** shows higher magnification of a 0.56 µm optical section of the area indicated by arrow. Note the tortuous vessel (arrow). Panel **b** denotes higher magnification of an artery (identified by * in panel **a**) with perivascular activated microglia (Iba1^+^, red). Panels **c**, **d** show GFAP^+^ intracellular material (green) in perivascular activated microglia (Iba1, red; GFAP, green). Scale bar, 200 µm **a**; 10 µm **b**–**d**

**Fig. 18:**
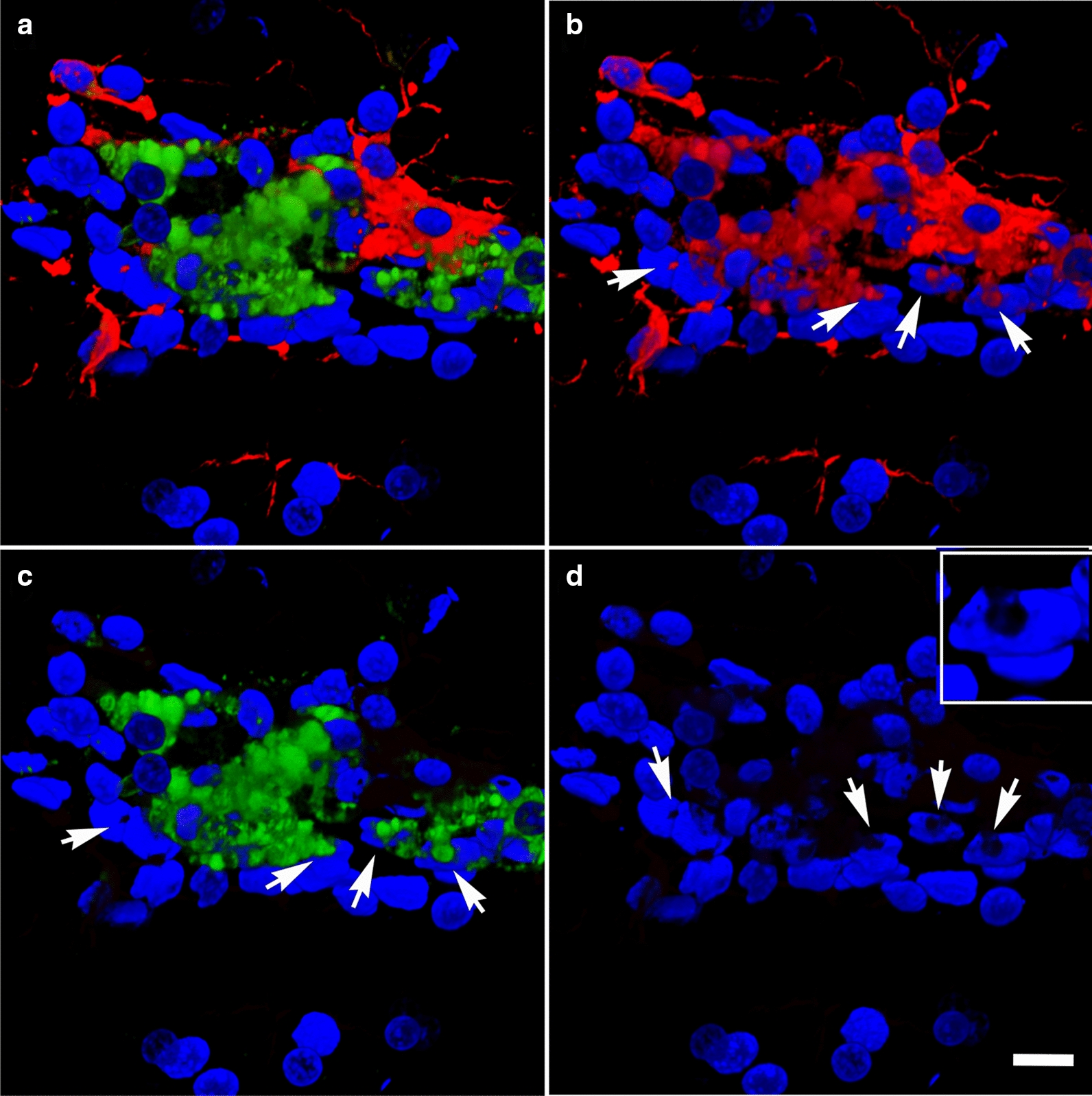
Perivascular microglial activation. MHCII expression is associated with microglial activation. Shown are perivascular amoeboid Iba1^+^ microglia (red). Some microglia are also expressing MHCII (green) and extend transcellular processes across the nuclei of Lochkern-like cells. **a**, Merged images, **b** Iba1 staining (red); **c** MHCII staining (green); and **d** DAPI staining (blue). Insert in **d** shows an enhanced view of a Lochkern-like cell. Arrows indicate the location of the Lochkern-like cells with hollow nuclei and the transcellular microglial processes (Iba1^+^ and MHCII^+^). Cellular blebs indicate that MHCII^+^ microglia appear to be undergoing apoptosis. Scale bar, 15 µm **a**–**d**; 5 μm insert in **d**

At 13 months post-blast, activated microglia were predominantly Iba1^+^ types 3 and 4 microglia (relative large soma and short ramifications) in areas neighboring affected vessels (Figs. 16, 17). Electron microscopy also showed that these vascular-associated microglia harbored dilated endoplasmic reticulum (Fig. 15e). Activated microglia tended to cluster together and were frequently observed in contact with the affected vasculature. Figure 16a–c shows a tortuous tract of the pericallosal artery within the corpus callosum with clustered Iba1^+^ activated microglia. Similarly, Fig. 17 also shows activated microglia next to affected vasculature. Evidence for perivascular microglial phagocytosis of astrocytes was obtained in confocal optical sections as intracellular GFAP^+^ material was found within perivascular Iba1^+^ activated microglia (Figs. 16d–f, 17c, d). Small perivascular patches of activated microglia expressing high levels of MHCII (MHCII^+^, characteristic of M1 microglia), were also observed in the brain of a blast-exposed animal. Some perivascular microglia were visibly undergoing apoptosis as seen by their TUNEL staining (Fig. 7) and presence of apoptotic blebs (Fig. 18). Furthermore, Iba1^+^ processes from activated perivascular microglia seemed to transverse the nucleus of neighboring Lochkern-like vascular cells (Fig. 18).

## Discussion

### A rat model of low-level blast exposure

We have been studying a rat model developed to mimic blast exposures associated with mild human TBI [4]. Because multiple blast exposures were common among veterans returning from Iraq and Afghanistan [31], we used a design in which rats received three blast exposures, delivered one per day on 3 consecutive days. Under the blast exposure conditions utilized here (74.5 kPa, 10.8 psi), there is no post-exposure apnea, mortality is rare and there are no pulmonary hemorrhages or other lung pathology. During the blast overpressure exposures head motion is restricted to minimize rotation/acceleration injury. Control (sham-exposed) animals are treated identically including receiving anesthesia and being placed in the blast tube, but are not blast-exposed. The lack of histological evidence for coup/contrecoup injuries or generalized brain tissue damage supports the mild nature of the injury and lack of significant rotation/acceleration injury [4, 30].

### Blast exposure affects the cerebral vasculature

**Fig. 19:**
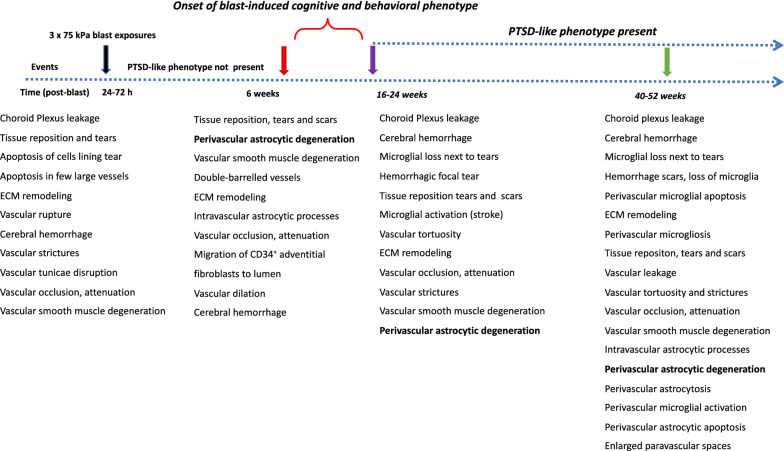
Summary timeline of cerebral pathological alterations in a rat model of blast-induced mild TBI. Shown are observations in blast-exposed animals from acute, subacute and chronic cohorts, all of which received 3 × 74.5 kPa exposures [2, 39–41, 63, 66, 67]. Acute, 24-72 h post-blast; Subacute, 6 weeks post-blast (normal behavioral phenotype); Chronic, 16–52 weeks post-blast, (PTSD-like behavioral phenotype)

The high metabolic demand of the brain requires tight coordination between neuronal activity and blood flow [10, 23, 29, 48, 58]. Because gliovascular and neurovascular interactions control cerebral blood flow at multiple levels, their disruption following blast exposure would be expected to affect cerebral autoregulation. Morphological, biochemical and functional studies of humans and animal models have identified the vasculature as a primary target for blast-induced tissue damage [2, 6, 32, 38–41, 45, 50, 54, 63, 66, 75, 82, 112–114, 116, 130–133, 138]. Considerable evidence supports the concept of a thoracic effect whereby pressure waves transmitted through the systemic circulation damage the brain providing an additional mechanism for why blood vessels and perivascular cellular elements may be particularly susceptible to blast injury [116, 117]. A summary timeline of cerebral pathological alterations in acute, subacute and chronic cohorts are shown in Fig. 19.

In the present study, we investigated the evolution of blast-induced vascular disease using a rat model. Notably, the general structure of the circulatory system of rats is comparable to that of humans, with the brain deriving its arterial blood supply from the extracranial carotid and vertebral arteries. The internal carotid arteries and their branches supply the anterior two-thirds of the cerebral hemispheres, including the deep white matter and basal ganglia. The vertebral arteries that fuse into the basilary artery and its branches supply the remaining posterior and medial regions of the hemispheres, most of the diencephalon, brainstem, cerebellum, and cervical spinal cord. Blast exposure leads to prolonged impairment in vasoreactivity as well as changes in the structure, integrity and phenotype of cerebral blood vessels and astrocytes [2, 40, 63, 66]. Localized blast-induced vascular injury may be a determinate of the brain areas where functional alterations will occur.

### Blast-induced vascular alterations

Micro-CT is becoming a widely used method for the accurate resolution of three-dimensional cerebral vascular structures and quantitative volumetric measurements [28, 145]. By applying this methodology in combination with computerized morphometry, we performed quantitative analyses of the brain vasculature of blast-exposed and control rats at different time points post-exposure reflecting acute, subacute and chronic periods.

Statistical analyses did not show significant differences in the vascular length, surface area and volume between blast-exposed and control groups at any time point when parameters were analyzed across the whole brain. However, micro-CT analysis allowed the visualization of regional blast-induced alterations of the cerebral vasculature. For example, a rat brain examined 48 h post-exposure showed vascular attenuation in the left hemisphere, affecting the hippocampus as well as the retrosplenial, visual, auditory, perirhinal and piriform cortical regions as well as part of the amygdala. This involved, most likely alterations in the anterior and posterior circulations, maybe through occlusion or attenuation of the posterior branches of the MCA as well as collateral branches of the PCA and the AChA. At 6 weeks and 13 months post-blast exposure, animals also presented regional alterations and disorganization in the cerebral vasculature. Lesions in the brain (tissue tears) associated with vascular alterations (vascular disorganization and hypoperfusion) correlated with behavioral observations as a rat with bilateral amygdalar lesions, cortical hypoperfusion and hippocampal vascular disorganization failed to express the characteristic chronic anxiety-related behaviors of blast-exposed animals.

We previously described blast-induced vascular attenuation/occlusion due to amorphous cellular debris, vascular collapse due to the negative pressure generated by the fast movement of blast-displaced blood, vascular “pinching” where opposite sides of the vascular walls adhere to each other, occlusion by CD34^+^ cells, tortuosity and structural alterations in the arterial muscle layer [3, 38, 40].

### Dissociation of the vasculature from the neuropil and vascular remodeling

Electron microscopy and morphological analyses of brain samples from the 13 months post-exposure cohort showed development of a unique pathology characterized by dissociation of arterioles from the brain parenchyma (neuropil) and perivascular astrocytic degeneration. The arterial/arteriolar wall is a three-layered structure composed of an intima (endothelial cells), media (smooth muscle cells) and adventitia (fibroblasts, nerves, progenitor and immune cells) [23, 87]. The adventitia is lined with astrocytic endfeet and serves as an anchor for nerve endings [36, 87, 129]. Each layer contributes in unique ways to mantaining vascular homeostasis and regulating vascular responses to environmental conditions, stress or injury [129]. The adventitia is the principal vascular injury sensing tissue in which local fibroblasts (mesenchymal progenitors) play a central role in vascular remodeling by modulating smooth muscle cells and interacting with endothelial cells through soluble factors [53, 87, 129]. Adventitial fibroblasts produce and organize elements of the ECM (fibrillar collagens type I and II, collagen type IV, fibronectin, tenascin, osteopontin, thrombospondin), which provide mechanical strength to the vessel [108, 127, 129]. They are also involved in vascular remodeling via the regulation of matrix metalloprotease degradation of the ECM through TIMPs [18, 78, 129, 144].

Acute increases in cerebral MMP-9 associated with blood–brain barrier disruptions have been observed 2–3 h to 1 day post-blast exposure [66]. Blast-induced brain injury, as well as other forms of traumatic brain injury, have shown biphasic patterns of blood–brain barrier disruptions, where the barrier re-establishes itself after the acute phase and reopens at a later point in time [9, 11, 55, 66, 81, 106, 119]. Our results show that blast injury induces a chronic remodelling of the arteriolar laminae as evidenced by increased levels of metalloproteinases MMP-2 and MMP-9 observed at 6 weeks post-blast as well as decreased levels of vascular collagen type IV in the affected vasculature at 13 months post-exposure.

Matrix metalloproteases are responsible for degradation of ECM components, including basement membrane collagen, interstitial collagen, fibronectin and various proteoglycans [129]. Smooth muscle cells produce metalloproteases [62, 88, 93]. Vascular infiltrating inflammatory cells, endothelial cells and fibroblasts are also known to produce metalloproteases [79]. Excessive expression of vascular MMP-2 and MMP-9 gelatinase activity decreases the elasticity and modifies the structure and mechanical properties of the vessel by degrading the elastic and collagenous fibers of the tunica media and adventitia in addition to the basal lamina.

Acutely, activation of metalloproteases induces vasoconstriction via the transactivation of epidermal growth factor receptors and the production of reactive oxygen species (ROS) [51, 87, 88, 94]. Chronically, metalloproteases and ROS can induce remodeling processes in response to prolonged vasoconstriction [87, 88]. The observed thickened basal and elastic laminae in the cerebral vasculature of blast-exposed animals could be a reflection of this phenomenon (Fig. 14). Altogether, these alterations affecting the composition and physical characteristics of the basal membrane associated with the arterial vascular smooth muscle would predict hypofunctionality of ISF flow via the IPAD system[5]. The presence of extravascular IgG also attests to vascular fragility resulting in leakage, which in turn may trigger more neuroinflammation and carry proteases involved in ECM degradation into the CNS from the periphery.

### Chronic astrocytic degeneration

Astrocytes are multifunctional brain cells that provide ionic and trophic support to neurons, regulate intracerebral circulation and have an important role in cognitive functioning [13, 72, 77, 85, 86, 92, 96, 135]. Perivascular astrocytic degeneration is visible at 72 h through 6 weeks post-blast with no overt alterations in neuronal somato-dendritc morphology [40, 81]. Similarly, decreased levels of GFAP have been observed in rats 24 h post-blast exposure [117]. Blast exposure induces epigenetic alterations with decreases in astrocytic histone acetylation and altered gene expression [8].

In our rat model, late chronic vascular alterations induced by blast exposures were previously documented at 6–10 months following exposure [32, 38–40]. In the present study at 13 months post-blast, astrocytic degeneration and perivascular apoptosis was observed to be associated with enlarged paravascular spaces and dissociation of the vasculature from the parenchymal neuropil. Perivascular astrocytic endfeet from the blast-exposed animals were swollen with degenerating organelles, including elongated and swollen mitochondria and cellular debris. It is known that mitochondrial swelling causes rupture of the outer membrane and release of pro-apoptotic proteins such as cytochrome c into the cytosol [103]. Immunohistochemical analyses and confocal microscopic observations showed evidence for phagocytosis of perivascular astrocytes by activated microglia (Figs. 16, 17).

At advanced degenerative stages, the vasculature was dislocated from the parenchyma as bridging perivascular astrocytes were lost, suggesting that blast induces chronic perivascular astrocytic degeneration. Several studies have documented astrogliosis following blast injury [8, 26, 27, 32, 43, 65, 98]. In addition, a distinct type of glial scar and cortical perivascular astrocytosis have been described in humans exposed to blast injury [122]. Our study shows widespread, blast-induced loss of perivascular astrocytes. At 13 months post-exposure, loss of perivascular astrocytic coverage was clearly visible in GFAP- and collagen IV-immunolabeled sections and confirmed by electron microscopic observations. The loss of perivascular astrocytes would affect CSF flow through the glymphatic system. The dramatic enlargement of the paravascular space associated with perivascular astrocytic loss in major regions of the brain at 13 months post-blast may also indicate the continuous expansion of initial blast-induced neurovascular disconnections across the vasculature through the mechanical action of heartbeat-induced arterial peristaltic pulsations [68]. Supporting a possible similar effect in humans, Piantino et al. [104] found evidence for enlarged perivascular spaces by MRI in living Iraq/Afghanistan veterans who had been exposed to blast. These enlarged perivascular spaces (termed PVS burden) had a significant positive correlation with number of blast-induced TBIs [104]. They were also associated with poor sleep and persistent post-concussive symptom severity. Thus, multiple lines of evidence in animals and humans support the notion that the perivascular space is disturbed and enlarged after blast injury.

One curious observation is the presence of intraluminal astrocytic processes within blood vessels (Fig. 10). This phenonenon has been repeatedly observed in this and previous studies [40]. We suspect that the intraluminal astrocytic processes appear in hypofunctional damaged vessels into which astrocytic cells and processes could extend. Based on earlier work by six weeks after blast exposure, there is loss of GFAP in isolated vascular fractions with swelling and degeneration of astrocytic endfeet, creating an effective gliovascular disconnection [40]. However, vascular fractions from animals taken at 8 months post-blast exposure show normal levels of GFAP, suggesting some attempt at restablishing astrocytic perivascular connections [40] although the connections remain highly abnormal (Fig. 6). The unusual accumulation of perivascular astrocytes in some affected vein-like vessels at 13 months post-exposure further supports a late-onset astrocytic recovery, that although abnormal is occuring in some vessels (Fig. 10).

Reductions in cerebral blood flow result in release of vasoactive metabolites that induce vasodilation [23]. Blood flow is also controlled by hypercapnia and hypoxia, which induce dilation of cerebral arteries and arterioles, resulting in increased blood flow. On the other hand, hypocapnia causes vasoconstriction and decreased blood flow. Arterial autoregulation of blood flow is further maintained by contraction of arterial smooth muscle, which is also disturbed in this model [40]. Unlike pial arteries and arterioles, parenchymal arterioles are in close association with astrocytes and to a lesser extent with neurons [23]. Cortical vessels are innervated from subcortical brain regions with the majority of neural varicosities targeting the astrocytic endfeet surrounding arterioles [74].

Although direct signaling from neurons to blood vessels contributes to the regulation of cerebral blood flow, astrocytes play an important role in mediating neurovascular coupling [7, 60, 70]. Glutamate released from neurons acts on astrocytic metabotropic glutamate receptors to evoke Ca^2+^-dependent release of vasoactive metabolites of arachidonic acid from astrocytic endfeet. These astrocytic mechanisms, regulated by pO_2_ and lactate, induce either vascular dilation via prostaglandin E2 (PGE2) and epoxyeicosatrienoic acids (EETs) or vasoconstriction via 20-hydroxyeicosatetraenoic acid (20-HETE). Under normoxic conditions, astrocytic Ca^2+^ signaling results in vasodilation. Under hyperoxia, however, vasoconstriction is favored. Release of K^+^ from astrocytic endfeet upon active neuronal stimulation may also contribute to vasodilation [35]. Direct stimulation of astrocytes raises calcium levels in the endfeet to induce the release of K^+^, 20-HETE and PGE2, which in turn causes dilation of neighboring arterioles [34, 90, 146].

Neuronal activity may also regulate capillary blood flow through Ca^2+^-dependent astrocytic mechanisms [15, 89] that actively dilate and constrict pericytes in response to vasoactive agents. Pericyte-induced relaxation has been shown to be mediated by activation of PGE2 receptors and by nitric oxide inhibition of the synthesis of the vasoconstrictor 20-HETE [47, 84]. Pericyte degeneration leads to neurovascular uncoupling and limits oxygen supply to the brain [71].

Control of local blood flow to match neuronal demand results, in part, from the release of neurotransmitters that stimulate receptors on smooth muscle, endothelium, pericytes or astrocytes to induce constriction or dilation through multiple signaling pathways [7, 23, 29, 48, 49, 58–60, 109, 121]. Therefore, any disruption within the neurovascular unit by blast exposure is likely to result in altered cerebral perfusion. As confirmed here, blast exposure results in structural alterations of the arterial medial smooth muscle layer. In addition to disrupted gliovascular and neurovascular signaling and cerebral circulation, glymphatic flow and IPAD drainage could also be impaired by altered vascular smooth muscle structure[5, 142]. In the human context failure of the glymphatic and IPAD systems may translate in the long term to Abeta deposition, development of cerebral amyloid angiopathy (CAA) and Alzheimer’s disease [5].

### Microglia activation associated with vascular degeneration

Microglia are small macrophage-like glial cells of the central nervous system that constitute ~ 10% of the cells in the brain [57, 76, 107]. They are in contact with synaptic elements (axon terminals, dendritic spines, astrocytic processes and synaptic clefts) where they maintain homeostasis, remove tissue debris and pathogens, and remodel synapses [16]. Upon neural injury, pathological states or chronic stress, microglia are activated and undergo morphological, proliferative and physiological changes that increase phagocytic activity and secretion of proinflammatory molecules [107]. This results in propagation of neuroinflammatory and neurodegenerative responses.

Microglial morphologies are associated with different states of activation and include ramified, primed, reactive, and ameboid microglia (Types 1–4, respectively) [69, 73, 120, 124, 128, 139]. Functional microglial activation states include the pro-inflammatory M1 activation associated with enhanced neurotoxicity and ECM damage, anti-inflammatory and neuroprotective M2a activation that induces phagocytosis, and the microglia-deactivating M2c activation involved in wound healing [143].

In a previous study we found no morphological or biochemical evidence of inflammation in the brains of rats 6 weeks post-blast exposure [41]. However, neuroinflammation was reported in an animal 16 weeks post-blast exposure with evidence of vascular rupture [41]. In the present study we present evidence that rats 13 months post-blast exposure exhibited neuroinflammation associated with vascular alterations including the presence of perivascular activated MHCII^+^ microglia. Expression of the major histocompatibility class II (MHCII) in microglia is linked to the inflammatory response in neurodegenerative diseases [118]. Our results indicate that this vascular-associated neuroinflammation develops after the subacute phase (6 weeks post-blast) into the chronic stage. In association with degenerating perivascular astrocytic endfeet, we also identified microglia with dilated endoplasmic reticulum, mitochondrial alterations and cholesterol crystals in lysosomal vacuoles. Cholesterol crystals have been shown to induce destabilization of lysosomes, causing leakage of the protease cathepsin B, leading to activation of the innate NLRP3 inflammasome complex, resulting in caspase-1–mediated activation and secretion of proinflammatory cytokines [44, 110]. Following blast-induced vascular fragility and leakage, cholesterol-rich debris (including myelin) may be engulfed by microglia and infiltrating macrophages into phagosomes, which fuse with lysosomes to form phagolysosomes. Compromised reverse cholesterol transport, which normally mediates efflux of cholesterol will result in cholesterol overload, leading to formation of lysosomal membrane-damaging cholesterol crystals, release of lysosomal enzymes, inflammasome activation and cytokine secretion [80].

It has been shown that local inflammation associated with vascular degeneration may be due to elevated permeability of the blood–brain barrier. This results in clustering of activated microglia which contact the affected vasculature [16, 24, 46, 52, 126]. The dilated endoplasmic reticulum identifies these cells as dark microglia [16, 126].

Interestingly, rare activated microglia neighboring blood vessels contained cells with hollow nuclei (Lochkern-like cells [12, 105]) through which microglial processes seemed to transverse. Although the significance of this unusual observation remains unknown, transcellular migration of microglia has been extensively described in the retina after neuronal damage [64, 95, 111, 137]. This process is involved in the rapid recruitment and migration of immune and inflammatory cells to inflammatory sites without the loss of barrier integrity. Microglial transcellular and paracellular transport within the neurovascular unit [52] may be increased by the chronic vascular degenerative events induced by blast exposure.

Microglia are also known to have a critical role in vascular repair and maintainance of vascular integrity in the central nervous system [46]. Chronic mild hypoxia in the spinal cord induces transient vascular leakage associated with activated microglia clustering next to the affected vasculature, particularly in the white matter. Microglial depletion in this system leads to exaggerated vascular leakage associated with astrocyte-vascular uncoupling and loss of endothelial tight junction proteins. Microglial vascular repair depends on fibrinogen–Mac1 receptor interactions [46]. Similarly, in our rat model of blast-induced traumatic brain injury, at 13 months post-exposure we observed clustered activated microglia next to affected vasculature in the brain white matter. Some of these cells exhibited intracellular GFAP^+^ material as evidence for microglial phagocytosis of astrocytes. TUNEL analysis showed perivascular apoptosis of astrocytes and astrocytic-associated microglia. The brains of blast-exposed animals at this late time point also presented vascular leakage as evidenced by the presence of extravascular IgG. Microglial repair function may prevent further major leakage. Apoptotic loss of perivascular microglia may result in loss of vascular repair and increased vascular fragility.

## Conclusions

Our results show that blast exposure affects cellular interactions within the neurovascular unit and that astrocytes play a central role bridging the vasculature and the parenchymal neuropil. The cognitive and behavioral alterations that develop following blast exposure in both humans and animal models [30, 31, 33, 83, 99, 102] may be caused by the resulting disruptions in blood circulation, glymphatic CSF flow and intramural periarterial ISF drainage that develop as the result of altered neurogliovascular structure, astrocytic degeneration, vascular remodeling, vascular smooth muscle degeneration, vascular fragility, vascular leakage and neuroinflammation. Furthermore, impaired microglial function may impede vascular repair and accelerate the neurovascular degenerative processes associated with blast-induced traumatic brain injury.

## Data Availability

The datasets generated during and/or analysed during the current study are available from the corresponding author on reasonable request.
